# Yiyi Fuzi Baijiang formula protects against DSS-induced colitis by orchestrating the gut barrier-microbiota-metabolism axis

**DOI:** 10.1186/s13020-026-01478-x

**Published:** 2026-07-23

**Authors:** Yin Bao, Qiang Ao, Meng Wang, Xiaoling Mao, Jun Zhu, Mingyue Zhang, Yang Chen, Hong Zhu, Jun Gao

**Affiliations:** 1 Department of Gynecologic Oncology, Jiangxi Clinical Research Center for Cancer, Jiangxi Cancer Hospital & Institute, The Second Affiliated Hospital of Nanchang Medical College Nanchang, Nanchang, 330036 China; 2 State Key Laboratory of Phytochemistry and Natural Medicines, Dalian Institute of Chemical Physics, Chinese Academy of Sciences, Dalian, 116023 China

**Keywords:** Yiyi Fuzi Baijiang formula, Inflammatory bowel disease, Colitis, Gut microbiota, Metabolomics, Network pharmacology

## Abstract

**Background:**

Inflammatory bowel disease (IBD) is a relapsing inflammatory disorder of the gastrointestinal tract with increasing global incidence. Current therapies are often limited by side effects, loss of efficacy, and high cost, underscoring the need for safer and more effective alternatives, particularly multi-target agents derived from natural products.

**Purpose:**

This study aimed to elucidate the protective mechanisms of Yiyi Fuzi Baijiang formula (YFB), a traditional Chinese medicine (TCM) formulation, against dextran sulfate sodium (DSS)-induced acute colitis, focusing on its systemic regulation of the gut barrier–microbiota–metabolism axis.

**Methods:**

We employed an integrated approach combining network pharmacology, UPLC-Q-TOF-MS/MS-based phytochemical analysis, in vivo evaluation in a DSS-induced colitis mouse model, 16S rRNA gene sequencing, and untargeted metabolomics to assess the effects of YFB and uncover its mechanisms of action.

**Results:**

Network pharmacology predicted, and experiments confirmed, that core YFB components (e.g., quercetin, kaempferol) act via IL-17, TNF, and NF-κB pathways. YFB administration dose-dependently improved disease activity index, colon shortening, and histopathology in colitis mice. It restored intestinal barrier integrity by upregulating ZO-1, Occludin, and MUC2, while suppressing pro-inflammatory cytokines (TNF-α, IL-6, IL-1β, IL-17A) and NF-κB activation. Critically, YFB promoted epithelial repair by restoring the expression of intestinal stem cell marker LGR5 and progenitor cell marker SOX9, and by normalizing the aberrant increase in endocrine cell marker CHGA. YFB treatment was associated with reversal of DSS-induced gut microbiota dysbiosis, restoration of diversity, enrichment of beneficial bacteria (e.g., Lachnospiraceae), and suppression of opportunistic pathogens (e.g., Enterobacteriaceae). Untargeted metabolomics showed that YFB treatment was associated with modulation of DSS-altered fecal metabolites (e.g., fatty acids, bile acids) and pathways such as "microbial metabolism in diverse environments".

**Conclusion:**

YFB, when administered concomitantly with DSS, protects against DSS-induced colitis via the synergistic effects of its multi-component system. Its mechanism entails systemic regulation of the gut barrier–microbiota–metabolism axis, involving suppression of NF-κB-driven inflammation, promotion of intestinal epithelial repair (via LGR5/SOX9/CHGA modulation), restoration of the intestinal barrier, alterations in gut microbiota, and modulation of host–microbial co-metabolism. These findings provide a scientific basis for YFB's clinical application and highlight the value of TCM formulations in managing complex multi-factorial diseases.

**Graphical Abstract:**

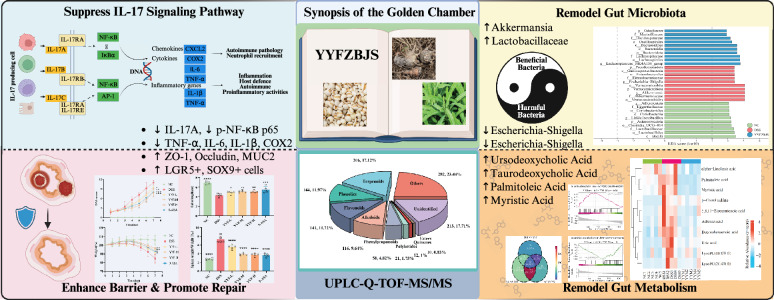

**Supplementary Information:**

The online version contains supplementary material available at 10.1186/s13020-026-01478-x.

## Introduction

Inflammatory bowel disease (IBD), a relapsing inflammatory disorder of the gastrointestinal tract, has exhibited a steadily rising global incidence over the past decade, posing a significant public health challenge [5, 7, 32]. Recent epidemiological studies describe its global progression through distinct stages: an initial phase of low incidence (Stage 1), followed by accelerated growth (Stage 2), and currently, a stage of compounded prevalence increase (Stage 3) [10]. Current mainstay therapies for IBD, including aminosalicylates, corticosteroids, immunosuppressants, and biologics [13], primarily aim to suppress excessive immune responses [14]. However, their long-term use is frequently hampered by notable adverse effects [38], diminishing efficacy over time (loss of response) [30], and substantial economic burden.

Furthermore, the emergence of immune checkpoint inhibitor (ICI)-associated colitis, a distinct and often severe form of IBD driven by overactivated T-cells in cancer patients receiving immunotherapy, underscores the complexity of IBD pathogenesis and the limitations of current immunosuppressive strategies [16, 33]. This clinical dilemma underscores the urgent need for safer, more effective, and cost-efficient alternative therapies, particularly multi-target agents derived from natural products [11, 20, 21, 24].

The pathogenesis of IBD is multifactorial, involving intestinal barrier dysfunction, gut microbiota dysbiosis, and host-microbe co-metabolic disturbances [13]. A hallmark of IBD is the impairment of the intestinal physical barrier, characterized by disrupted expression of tight junction proteins such as ZO-1 and occludin [44]. This breach facilitates bacterial translocation, perpetuating a cycle of inflammation [35–37] and exacerbating microbial dysbiosis [28]. These microbial alterations further drive dysregulation of microbially derived metabolites, such as short-chain fatty acids [42] and bile acids [3], which are crucial for maintaining immune homeostasis. These three elements—barrier integrity, microbiota composition, and the metabolic landscape—are intricately interconnected, forming the “gut barrier–microbiota–metabolism” axis. This axis drives disease progression via key signaling pathways, including IL-17 and NF-κB [8]. Consequently, protective strategies capable of simultaneously targeting this interconnected axis hold great promise for IBD management.

In this therapeutic context, Traditional Chinese Medicine (TCM) formulations, characterized by their multi-component, multi-target, and synergistic nature, offer distinct advantages. Among them, the Yiyi Fuzi Baijiang formula (YFB), derived from the classical text *Jin Gui Yao Lue* (Synopsis of the Golden Chamber), has been traditionally used to treat “intestinal abscess,” a condition whose manifestations closely resemble those of modern inflammatory bowel disease (IBD). The formula comprises three medicinal components: Coicis Semen, Aconiti Lateralis Radix Praeparata, and Patriniae Herba.

Pharmacological studies have begun to elucidate the active constituents and mechanisms of individual herbs: polysaccharides and lipids from Coicis Semen show anti-inflammatory effects and ameliorate immune dysfunction in murine colitis [43], alkaloids from Aconiti Lateralis Radix Praeparata can modulate macrophage polarization and alleviate systemic inflammation [31], and Patriniae Herba has demonstrated potential to modulate gut microbiota, along with anti-inflammatory and anti-tumor activities [15, 35–37]. Investigations into the composite formula YFB have identified its regulatory effects on classical inflammatory pathways in ulcerative colitis [21, 24] and colorectal cancer [34, 41]. However, existing research has largely been confined to macroscopic pharmacodynamic observations and validation of isolated pathways, failing to elucidate the synergistic, multi-component, multi-target interactions that are central to TCM theory. Substantial knowledge gaps remain, particularly concerning its effects on intestinal barrier repair, microbiota interactions, and the cooperative mechanisms underlying its multi-target actions. Specifically, whether and how YFB exerts a systematic regulatory effect on the “gut barrier–microbiota–metabolism” axis is a critical and unresolved question.

Therefore, this study was designed to address these gaps by systematically investigating the holistic mechanism of YFB against colitis. We integrated modern analytical techniques with experimental and computational approaches. Specifically, we first used UPLC-Q-TOF-MS/MS to characterize its chemical composition [6]. We then established a DSS-induced acute colitis mouse model to evaluate protective effects via disease activity index, colon length, histopathology, and tight junction protein expression. Finally, we integrated 16S rRNA gene sequencing and untargeted metabolomics to investigate its effects on gut microbiota structure and host-microbial co-metabolism. This work not only provides a scientific basis for the clinical application of YFB but also illustrates the value of TCM formulas in treating complex diseases through multi-target synergy, following a comprehensive research chain from chemical constituents to pharmacodynamic evaluation and mechanistic exploration. Furthermore, it offers insights for developing novel therapies targeting the gut barrier–microbiota–metabolism axis.

## Materials and methods

### Network pharmacology analysis of YFB for IBD

#### Screening of YFB compounds and target prediction

Potential active components of YFB and their targets were retrieved from the Traditional Chinese Medicine Systems Pharmacology Database (*TCMSP*), the Herbal Encyclopedia Resource (*HERB*), and the Traditional Chinese Medicine Integrated Database (*TCMID*). Components were screened based on oral bioavailability (OB ≥ 30%) and drug-likeness (DL ≥ 0.18) [27]. Corresponding gene names were standardized using the *UniProt* database [12]. A herb-compound-target network was then visualized using *Cytoscape* (version 3.7.2) [9].

#### Analysis of differentially expressed genes in IBD patients

Differentially expressed genes associated with human IBD were obtained from the *GeneCards* and *OMIM* databases. Genes with a *p* < 0.05 and an absolute log₂ fold change (|log₂FC|) > 1 were considered significant.

#### Construction of compound-target network and protein–protein interaction network

Overlapping genes between YFB candidate targets and IBD-associated genes were identified using Venn diagrams and visualized in *Cytoscape*. Potential YFB targets were imported into the *STRING* database to construct a protein–protein interaction (PPI) network [23]. The *CytoNCA* plugin in *Cytoscape* was then used to calculate six topological parameters (Betweenness, Closeness, Degree, Eigenvector, LAC, and Network) for each node. Hub targets were identified through stepwise filtering, retaining nodes with parameter values above the median at each step.

#### Functional enrichment analysis

Gene Ontology (GO) enrichment and Kyoto Encyclopedia of Genes and Genomes (KEGG) pathway analyses were performed using the R packages “*clusterProfiler*” and “*org.Hs.eg.db*.” A significance threshold of *p* < 0.01 and *q* < 0.05 was applied. A compound-target-pathway network was visualized with Cytoscape.

### Preparation of YFB

Yiyi Fuzi Baijiang formula granules, consisting of Coix Seed (30 g; batch AB520793), Aconite Root (6 g; batch AB48A473), and Patrinia Scabiosaefolia (15 g; batch AB43A193), were obtained from Guangdong Yifang Pharmaceutical Co., Ltd. According to the manufacturer, the extraction yields (raw herb to granules) for the individual herbs are: Coix Seed 5:1, Patrinia Scabiosaefolia 7.5:1, and Aconite Root 10:1 (the yields vary by region, and these values are based on the product sold in Nanchang). To prepare the stock solution (1.33 g crude drug equivalent/mL), 51 g of granules were dissolved in pure water and adjusted to a final volume of 38.3 mL. The solution was sterilized by filtration (0.22 μm), aliquoted, and stored at -20 °C.

### UPLC-Q-TOF-MS/MS analysis of YFB chemical composition

Chromatographic separation was performed using a *Thermo Vanquish* UHPLC system (Thermo Fisher Scientific, USA) equipped with an *ACQUITY UPLC*^*®*^* HSS T3* column (2.1 × 100 mm, 1.8 μm; Waters, USA) maintained at 40 °C. For positive ion mode analysis, the mobile phase consisted of solvent A (0.1% formic acid (64-18-6, TCI, Tokyo, Japan) in water, v/v) and solvent B (0.1% formic acid in acetonitrile (75-05-8, Thermo Fisher Scientific, Massachusetts, USA), v/v). For negative ion mode analysis, solvent A (5 mM ammonium formate (540-69-2, Sigma-Aldrich, Missouri, USA**)** in water) and solvent B (acetonitrile) were employed. All aqueous solutions were prepared using ultrapure water generated by a Milli-Q system (Millipore, USA). A gradient elution program was implemented at a flow rate of 0.3 mL/min as follows: 0–1 min, 8% B; 1–8 min, 8–98% B; 8–10 min, 98% B; 10–10.1 min, 98–8% B; 10.1–12 min, 8% B. The injection volume was 2 μL.

Mass spectrometric analysis was conducted on a *Q Exactive*^*™*^ hybrid quadrupole-Orbitrap mass spectrometer (Thermo Fisher Scientific, USA) equipped with an electrospray ionization (ESI) source operating in both positive and negative ionization modes. Data acquisition was performed in full MS and data-dependent MS/MS (dd-MS^2^) mode. The instrumental parameters were optimized as follows: spray voltage, ± 3.50 kV; sheath gas pressure, 40 arb; auxiliary gas flow rate, 10 arb; capillary temperature, 325 °C; full MS scan range, m/z 100–1000; MS^1^ resolution, 70,000 FWHM; dd-MS^2^ resolution, 17,500 FWHM; normalized collision energy, 30 eV. The top 10 most intense ions from each MS^1^ scan were selected for fragmentation, with dynamic exclusion enabled. All data were acquired and processed using *Thermo Xcalibur* software (version 4.0).

### Animal experiments

#### Animals

Male C57BL/6 mice (6–8 weeks old) were obtained from GemPharmatech Co., Ltd. and housed under specific pathogen-free conditions in the animal facility of the Ganjiang Chinese Medicine Innovation Center. Mice were kept on a 12 h light/dark cycle with free access to standard chow and water.

#### Establishment and treatment of the IBD model

Mice were randomly divided into six groups (n = 5): Control (normal water), DSS (3% DSS in drinking water), YFB-L (DSS + YFB 3.325 g/kg), YFB-M (DSS + YFB 6.65 g/kg), YFB-H (DSS + YFB 13.3 g/kg), and 5-ASA (DSS + 5-ASA 200 mg/kg). Acute colitis was induced by providing 3% DSS ad libitum in drinking water for 7 days to all groups except the Control. Concomitantly, YFB and 5-ASA groups received daily oral gavage of their respective treatments (0.1 mL volume) for the 7-day period. This concomitant administration design was chosen to evaluate the protective effects of YFB against DSS-induced colitis. The medium dose of YFB (6.65 g crude drug/kg) was equivalent to the human clinical dose based on body surface area conversion.

### Assessment of colitis symptoms

Body weight and disease activity index (DAI) were recorded daily. DAI was scored as previously described (score range 0–12) [45]. On day 8, mice were euthanized. Colon length and spleen weight were measured immediately. Serum, colon tissue, and vital organs (heart, liver, kidney) were collected. Successful model induction was confirmed by a DAI score ≥ 6 and a > 20% reduction in colon length in the DSS group compared to the Control.

### Immunohistochemistry (IHC)

Colon tissues were fixed, paraffin-embedded, and sectioned at 4 μm. After deparaffinization and rehydration, antigen retrieval was performed in citrate buffer. Sections were incubated with primary antibodies at 4 °C overnight, followed by HRP-conjugated secondary antibodies for 1 h at room temperature. Diaminobenzidine (DAB) was used for chromogenic detection, and hematoxylin for counterstaining. Slides were imaged using a light microscope. Antibody information is listed in Table 1. Immunohistochemistry staining intensity was quantified using ImageJ software.

**Table 1:** Primary antibodies used for immunohistochemistry

Catalog number	Antibody name/Target	Dilution	Company (location)
17590-1-AP	TNF-alpha polyclonal antibody	1:1000	Wuhan San Yin, Wuhan, China
82335-1-RR	Phospho-NF-κB p65 (Ser468) recombinant antibody	1:600	Wuhan San Yin, Wuhan, China
27260-1-AP	Occludin polyclonal antibody	10:3000	Wuhan San Yin, Wuhan, China
21773-1-AP	ZO-1 polyclonal antibody	1:2000	Wuhan San Yin, Wuhan, China
30007-1-AP	LGR5 polyclonal antibody	1:200	Wuhan San Yin, Wuhan, China
10529-1-AP	Chromogranin A polyclonal antibody	1:1000	Wuhan San Yin, Wuhan, China
13393-1-AP	COX-1/Cyclooxygenase-1 polyclonal antibody	1:1000	Wuhan San Yin, Wuhan, China
12375-1-AP	COX2/Cyclooxygenase 2/ PTGS2 polyclonal antibody	1:100	Wuhan San Yin, Wuhan, China
27675-1-AP	MUC2 polyclonal antibody	1:2000	Wuhan San Yin, Wuhan, China
55152-1-AP	SOX9 rabbit mAb	1:200	Biyinilai, Shanghai, China

### Histological analysis

Colon tissues were fixed in 4% paraformaldehyde for 72 h, then processed routinely for paraffin embedding. Sections (4 μm) were stained with hematoxylin and eosin (H&E) or periodic acid–Schiff (PAS).

For H&E, sections were deparaffinized, rehydrated, stained with hematoxylin (5 min), blued, counterstained with eosin (3 min), dehydrated, cleared, and mounted.

For PAS staining, sections were oxidized with 0.5% periodic acid (10 min), rinsed, treated with Schiff reagent (15 min, dark), rinsed again, and counterstained with hematoxylin before dehydration and mounting.

Histopathological scoring was performed blinded by two independent researchers. Colitis severity was assessed based on inflammatory cell infiltration, mucosal architecture damage, and crypt morphology. Goblet cell number and mucus secretion were quantified from PAS-stained images using an image analysis system.

### Quantitative real-time PCR (RT-qPCR)

Total RNA was extracted from colon tissues with TRIzol reagent. cDNA was synthesized from 1 μg RNA using the PrimeScript RT Kit. RT-qPCR was performed with SYBR Green Master Mix on a QuantStudio system. Cycling conditions were: 95 °C for 30 s; then 40 cycles of 95 °C for 15 s, 60 °C for 30 s, and 72 °C for 20 s. *Gapdh* was the endogenous control. Relative expression was calculated by the 2^(−ΔΔCt)^ method. Primer sequences are in Table 2.

**Table 2:** Primer sequences used for RT-PCR in this study

Gene (mouse)	Forward (5'–3')	Reverse (5′–3′)
COX2	TTCAACACACTCTATCACTGGC	AGAAGCGTTTGCGGTACTCAT
MUC2	AGGGCTCGGAACTCCAGAAA	CCAGGGAATCGGTAGACATCG
TNF-α	CCCTCACACTCAGATCATCTTCT	GCTACGACGTGGGCTACAG
IL-1β	GCAACTGTTCCTGAACTCAACT	ATCTTTTGGGGTCCGTCAACT
IL-6	TAGTCCTTCCTACCCCAATTTCC	TTGGTCCTTAGCCACTCCTTC
IL-17A	TTTAACTCCCTTGGCGCAAAA	CTTTCCCTCCGCATTGACAC
CXCL2	CCAACCACCAGGCTACAGG	GCGTCACACTCAAGCTCTG
GAPDH	AGGTCGGTGTGAACGGATTTG	TGTAGACCATGTAGTTGAGGTCA

Annealing temperature: 60 ℃ for all qPCR reactions

### Enzyme-linked immunosorbent assay (ELISA) detection

Levels of TNF-α, IL-6, IL-1β, and IL-17A in colon homogenates and serum were measured using commercial ELISA kits. Colon tissues were homogenized in ice-cold PBS, and supernatants were collected after centrifugation. Protein concentration in homogenates was determined with a BCA assay kit for normalization. Absorbance at 450 nm was read on a microplate reader, and concentrations were interpolated from standard curves.

### 16S rRNA gene sequencing and analysis

Fecal DNA was extracted using a commercial kit. The V3–V4 region of the bacterial 16S rRNA gene was amplified with primers 338F and 806R. PCR products were checked, purified, quantified, and used to construct sequencing libraries. Paired-end sequencing (2 × 250 bp) was performed on an Illumina NovaSeq platform.

Raw sequences were demultiplexed, quality-filtered, merged, and checked for chimeras. Amplicon sequence variants (ASVs) were generated with DADA2. Taxonomy was assigned against the SILVA database (v138). Alpha diversity (Chao1, Shannon) and beta diversity (Bray–Curtis distance, PCoA, NMDS) analyses were performed in *QIIME2*.

### Untargeted metabolomics analysis

Fecal metabolites were extracted with 50% methanol, vortexed (1 min), incubated (10 min, RT), and centrifuged (4000 × g, 20 min). The supernatant was analyzed by UPLC coupled to a TripleTOF 5600plus high-resolution mass spectrometer. A pooled quality control sample was run intermittently. Raw data were processed using *XCMS* for feature extraction and alignment.

### Statistical analysis

Data are presented as mean ± SD. Statistical analysis was performed using *GraphPad Prism* (v9.0). Comparisons between two groups used Student’s t-test. For multiple groups, one-way ANOVA with appropriate post hoc tests was applied. *p* < 0.05 was considered statistically significant.

## Results

### Network pharmacology predicts core targets and pathways of YFB for colitis treatment

This study adopted a research strategy combining systems pharmacology and bioinformatics to explore the potential active components and their mechanisms of action of YFB in the treatment of immune intestinal diseases.

To identify the potential mechanisms of Yiyi Fuzi Baijiang formula (YFB) against colitis, we employed an integrated network pharmacology approach.

**Fig. 1:**
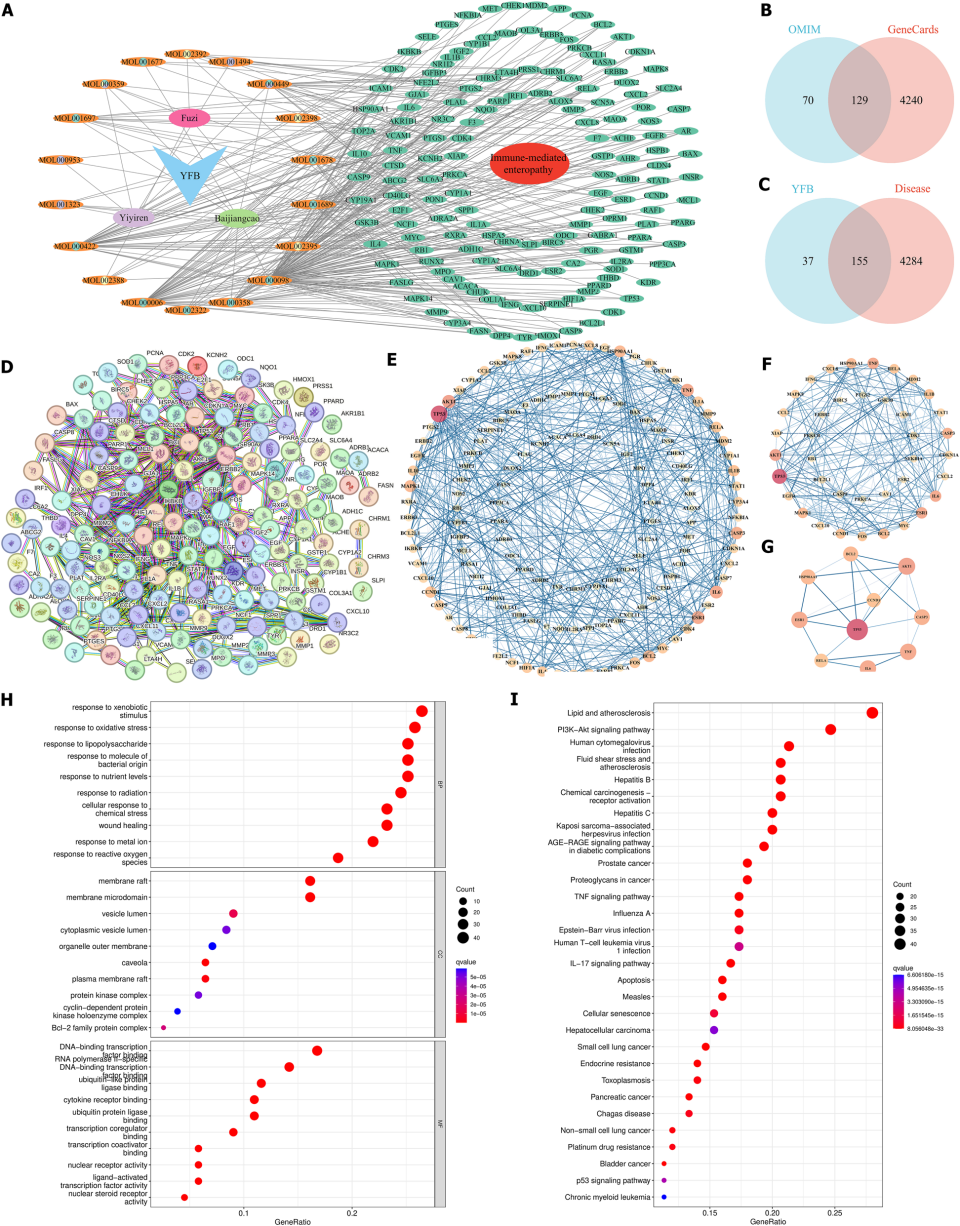
Network pharmacology analysis of Yiyi Fuzi Baijiang formula (YFB) for inflammatory bowel disease (IBD). **A** Herb-compound-target interaction network. Circular, orange-yellow, and green nodes represent herbs, compounds, and targets, respectively. **B** Venn diagram of IBD-related targets from *OMIM* and *GeneCards*. **C** Venn diagram showing the intersection between predicted YFB targets and IBD-associated targets. **D** Protein–protein interaction (PPI) networks after sequential screening: **E** initial network (142 nodes, 479 edges), **F** intermediate network (39 nodes, 182 edges), and **G** core hub network (10 nodes, 74 edges). Node size and color intensity correlate with connectivity (degree). **H** Gene Ontology (GO) enrichment analysis of the overlapping targets. **I** Kyoto Encyclopedia of Genes and Genomes (KEGG) pathway enrichment analysis. The size and color of bubbles represent the gene count and significance level, respectively

Screening based on oral bioavailability and drug-likeness yielded 142 potential active compounds from YFB, corresponding to 192 protein targets (*TCMSP*, *HERB*, *TCMID* databases). A herb-compound-target network was visualized (Fig. 1A). Meanwhile, 4,439 IBD-associated genes were retrieved from *GeneCards* and *OMIM* (Fig. 1B). Intersection analysis identified 155 overlapping targets as potential mediators of YFB’s effects in IBD (Fig. 1C).

To elucidate key interactions, a protein–protein interaction (PPI) network was constructed from the 155 overlapping targets using the *STRING* database (confidence > 0.9) and visualized in *Cytoscape* (Fig. 1D, E). Sequential filtering based on topology centrality parameters (Betweenness, Closeness, Degree, etc.) refined the network first to 39 core nodes (Fig. 1F), and then to a hub network of 10 nodes with 74 edges (Fig. 1G). The detailed topological parameters of the two screenings are provided in Supplementary Tables S1-S3. This hub network comprised BCL2, HSP90AA1, AKT1, CCND1, ESR1, TP53, CASP3, RELA, TNF, and IL6, representing the top predicted core targets of YFB for IBD.

Functional enrichment analysis of the 155 targets was performed. Gene Ontology (GO) terms were significantly enriched for biological processes including response to lipopolysaccharide and oxidative stress, and molecular functions such as cytokine receptor binding (Fig. 1H), suggesting anti-inflammatory and immunomodulatory potentials. Kyoto Encyclopedia of Genes and Genomes (KEGG) pathway analysis revealed strong enrichment in pathways critically involved in IBD pathogenesis, most notably the IL-17, TNF, and AGE-RAGE signaling pathways (Fig. 1I). Other enriched pathways included apoptosis, PI3K-Akt, and various cancer-related pathways, indicating a broad, multi-target mechanism of action.

### Identification of IL-17 pathway-related constituents in YFB by UPLC-Q-TOF-MS/MS

**Fig. 2:**
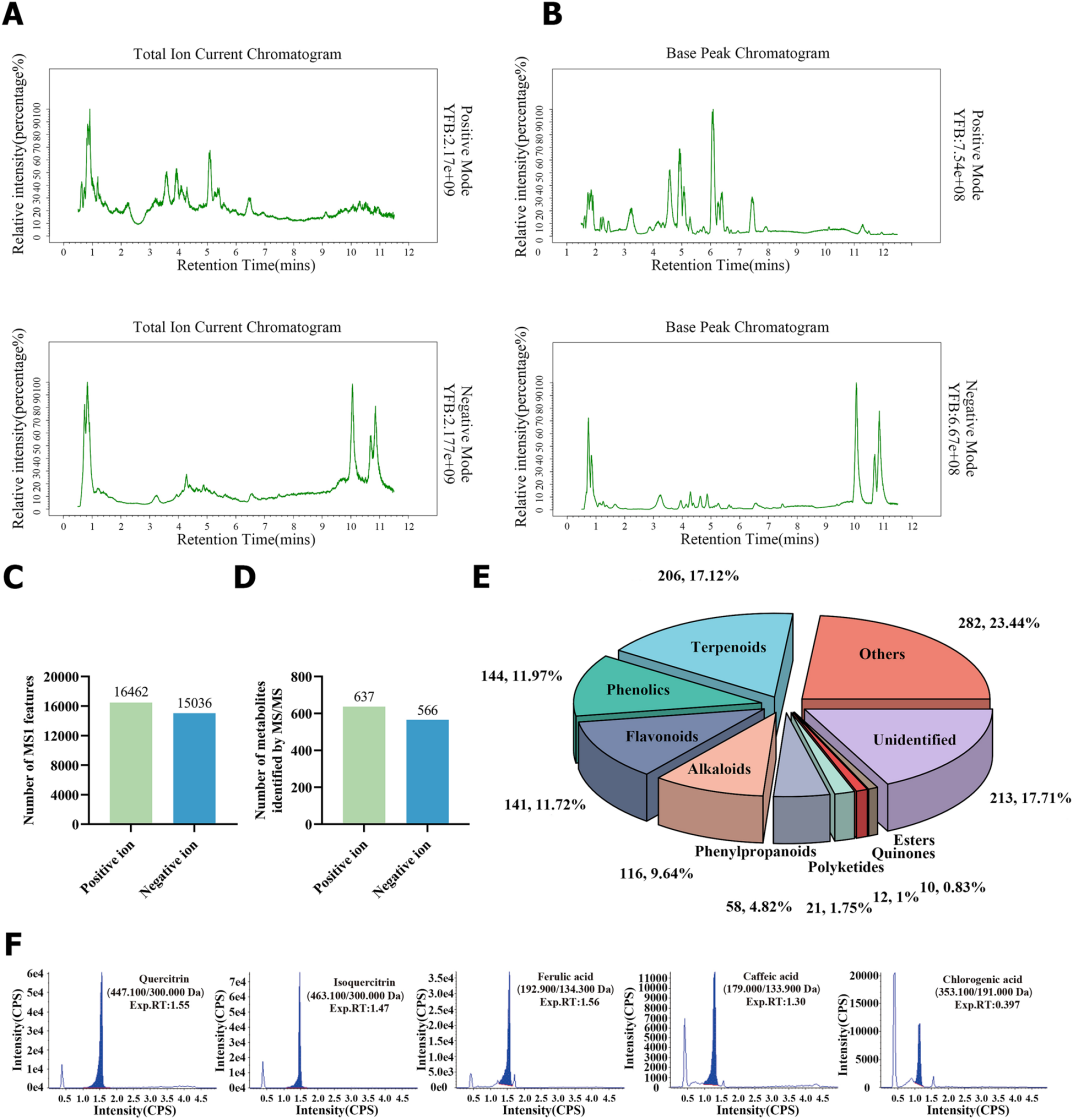
UPLC-Q-TOF-MS/MS analysis of Yiyi Fuzi Baijiang formula (YFB). **A** Total ion chromatograms (TIC) in positive and negative ion modes. **B** Base peak intensity (BPI) chromatograms. **C** Number of MS1 features and identified metabolites in each ion mode. **D** Distribution of major chemical classes identified in YFB. **E** Percentage distribution of different classes of metabolites identified in YFB. **F** Representative mass spectra of five major components (isoquercitrin, quercitrin, chlorogenic acid, caffeic acid, and ferulic acid) in YFB (Intensity, cps)

To bridge network pharmacology predictions with chemical reality, we performed a comprehensive UPLC-Q-TOF-MS/MS analysis of YFB. The total ion chromatograms (TIC) and base peak intensity (BPI) chromatograms showed good stability and component separation in both ionization modes (Fig. 2A, B). We detected 16,462 and 15,036 MS1 features in positive and negative modes, respectively, leading to the identification of 1,203 metabolites (Fig. 2C, D). Classification indicated that flavonoids, terpenoids, phenolics, and alkaloids were the major chemical classes (Fig. 2E).

Consistent with the network pharmacology results (Fig. 1A), the top-connected compounds (e.g., quercetin, kaempferol) were predicted to target the IL-17 pathway. UPLC-Q-TOF-MS/MS analysis confirmed the presence of these and other flavonoids in YFB. We identified multiple quercetin and kaempferol derivatives, along with acacetin and isovitexin, which are reported to be associated with anti-inflammatory activities (Table 3). Many of these compounds showed favorable predicted oral bioavailability (OB ≥ 30%) and drug-likeness (DL ≥ 0.18), and were detected at substantial levels, supporting their potential as key bioactive constituents of YFB.

**Table 3:** Representative flavonoids identified in Yiyi Fuzi Baijiang formula (YFB) by UPLC-Q-TOF-MS/MS

Category	OB (%)	DL	RT (min)	Observed m/z	Molecular formula	Identification
Quercetin	–	–	4.3	755.1998	C₃₃H₄₀O₂₀	Quercetin 3-neohesperidoside-7-rhamnoside
	–	–	4.3	757.1741	C₃₉H₃₂O₁₆	Quercetin 3-(3',6'-di-p-coumarylglucoside)
	–	–	4.4	521.1254	C₂₃H₂₄O₁₁	Quercetin 7,3,4-trimethyl ether 3-alpha-L-arabinopyranoside
	–	–	10.6	760.2317	C₃₂H₃₈O₂₀	Quercetin 3-(2Gal-apiosylrobinobioside)
	–	–	11.4	593.1506	C₂₇H₃₀O₁₆	Quercetin 3-galactoside 7-rhamnoside
Kaempferol	–	–	1.2	299.0544	C₁₆H₁₂O₆	3-O-Methylkaempferol
	–	–	4.4	287.0572	C₁₅H₁₀O₆	Kaempferol (aglycone)
	–	–	4.4	741.1896	C₃₉H₃₂O₁₅	Kaempferol 3-(2',6'-di-(E)-p-coumarylglucoside)
	–	–	4.9	903.2724	C₃₉H₅₀O₂₄	Kaempferol 3-[glucosyl-(1- > 3)-rhamnosyl-(1- > 2)-[rhamnosyl-(1- > 6)-galactoside]]
	–	–	4.3	801.2034	C_33_H_40_O_20_	Kaempferol 3-(6'-rhamnosylsophoroside)
	–	–	5.1	885.2741	C_39_H_50_O_23_	Kaempferol 3-isorhamninoside-7-rhamnoside
Acacetin	34.97	0.24	5.7	469.1087	C_24_H_24_O_11_	Acacetin 7-(6-acetylglucoside)
Isovitexin	31.29	0.72	4.4	739.1879	C₃₆H₃₆O₁₇	Isovitexin 2'-(6'-p-coumaroylglucoside)

“–” indicates that OB and DL values for glycoside derivatives were not available from the public databases (TCMSP, HERB, etc.)

Notably, beyond simple glycosides, we identified structurally modified flavonoids, including acylged glycosides (e.g., quercetin-3-(2',6'-di-p-coumaroylglucoside)) and methylated derivatives (e.g., quercetin-7,3,4-trimethyl ether) (Supplementary Table S1). Such modifications are known to improve bioavailability and metabolic stability compared to their parent aglycones. This suggests that YFB naturally contains “pre-optimized” forms of flavonoids, which may contribute to its potent in vivo efficacy.

Five major components in YFB were quantified by UPLC-MS/MS (Table 4). Caffeic acid (364 μg/g) and chlorogenic acid (44.9 μg/g) were the most abundant. Quercitrin (0.777 μg/g) and isoquercitrin (0.523 μg/g) were the major quercetin glycosides. Ferulic acid was 6.94 μg/g. Kaempferol aglycone was not detected, but multiple kaempferol glycosides were identified in Table 3, suggesting kaempferol may exist mainly as glycosides in YFB.

**Table 4:** Quantitative analysis of major phenolic acids and flavonoids in YFB (μg/g)

Compound	Content (μg/g)
Isoquercitrin	0.523
Quercitrin	0.777
Chlorogenic acid	44.9
Caffeic acid	364
Ferulic acid	6.94

### YFB protects against DSS-induced acute colitis in mice

**Fig. 3:**
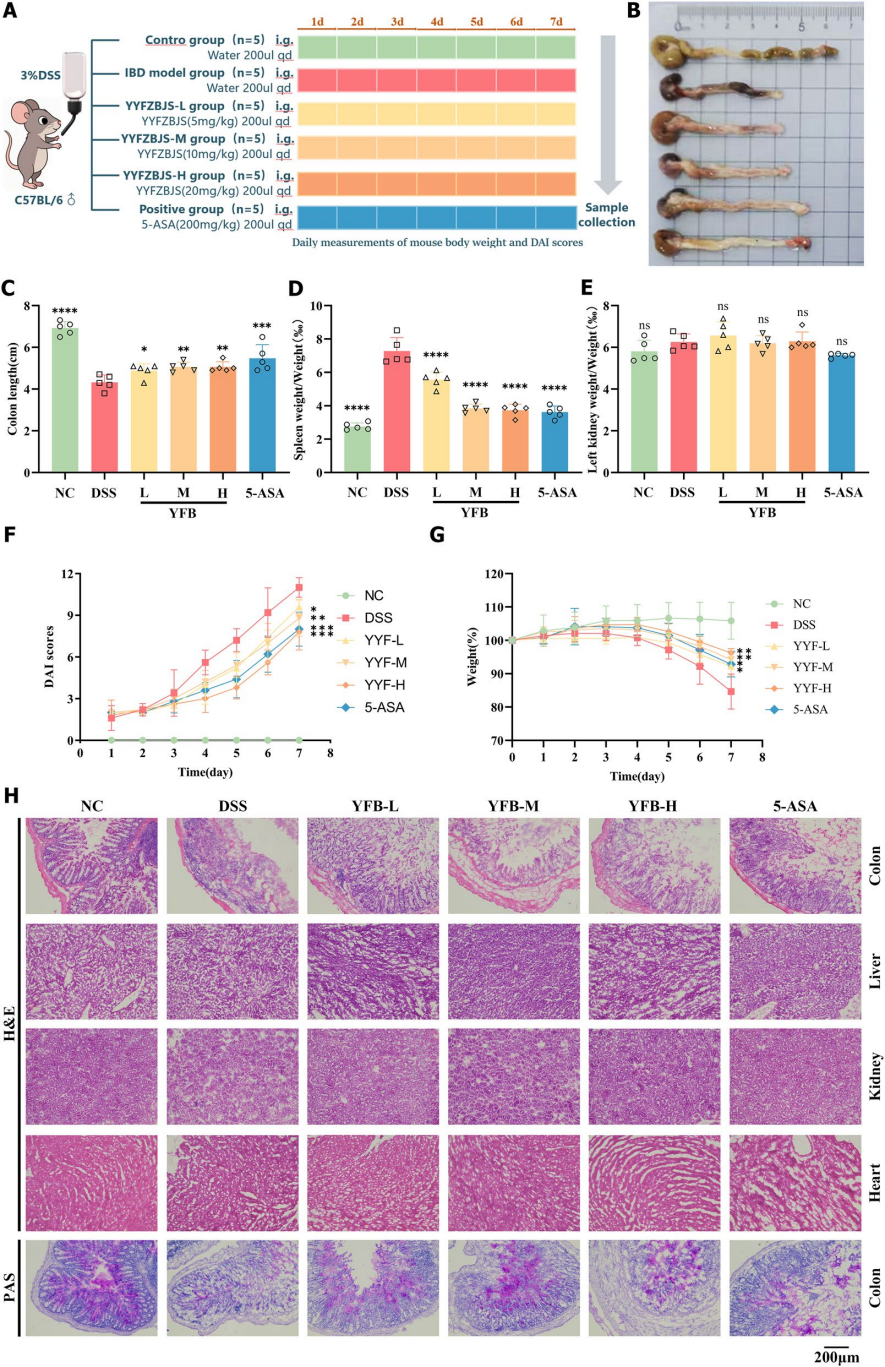
YFB protects against DSS-induced acute colitis in mice. **A** Experimental timeline. **B** Representative images of colons. **C** Colon length. **D** Spleen index. **E** Left kidney index. **F** Disease activity index (DAI). **G** Body weight change. **H** Representative H&E-stained images of colon, heart, liver, and kidney sections, and PAS-stained colon sections. Data are mean ± SD (n = 5). One-way ANOVA with post hoc test was used for panels **C**–**G**. *p* > 0.05(ns), *p* < 0.05 (*), *p* < 0.01 (**), *p* < 0.001 (***),* p* < 0.0001 (****) vs. DSS group. *NC* normal control, *DSS* DSS model, *YFB-L/M/H* YFB low/medium/high dose, *5-ASA* positive control

The protective effect of YFB was evaluated in a DSS-induced acute colitis mouse model (Fig. 3A). DSS administration caused significant body weight loss, increased disease activity index (DAI), colon shortening, and elevated spleen index compared to the normal control (NC) group. Treatment with YFB (3.325, 6.65, 13.3 g/kg) dose-dependently reversed these changes, attenuating colon shortening (Fig. 3B, C), reducing the spleen index (Fig. 3D) and left kidney index (Fig. 3E), improving DAI scores (Fig. 3F), and ameliorating weight loss (Fig. 3G). The medium and high doses of YFB showed efficacy comparable to the positive control, 5-aminosalicylic acid (5-ASA).

Histological assessment of colon tissue (H&E staining) confirmed severe damage in the DSS group, characterized by extensive inflammatory infiltration, crypt distortion, and epithelial damage (Fig. 3H). Both 5-ASA and YFB treatment groups showed markedly attenuated pathological changes, with better-preserved mucosal architecture and reduced inflammation. PAS staining further revealed that YFB prevented the DSS-induced loss of goblet cells and mucin depletion (Fig. 3H). Importantly, H&E staining of heart, liver, and kidney tissues from YFB-treated mice revealed no obvious pathological alterations, suggesting no acute toxicity at the tested doses.

### YFB suppresses inflammation, restores barrier integrity, and promotes epithelial repair in colitis mice

**Fig. 4:**
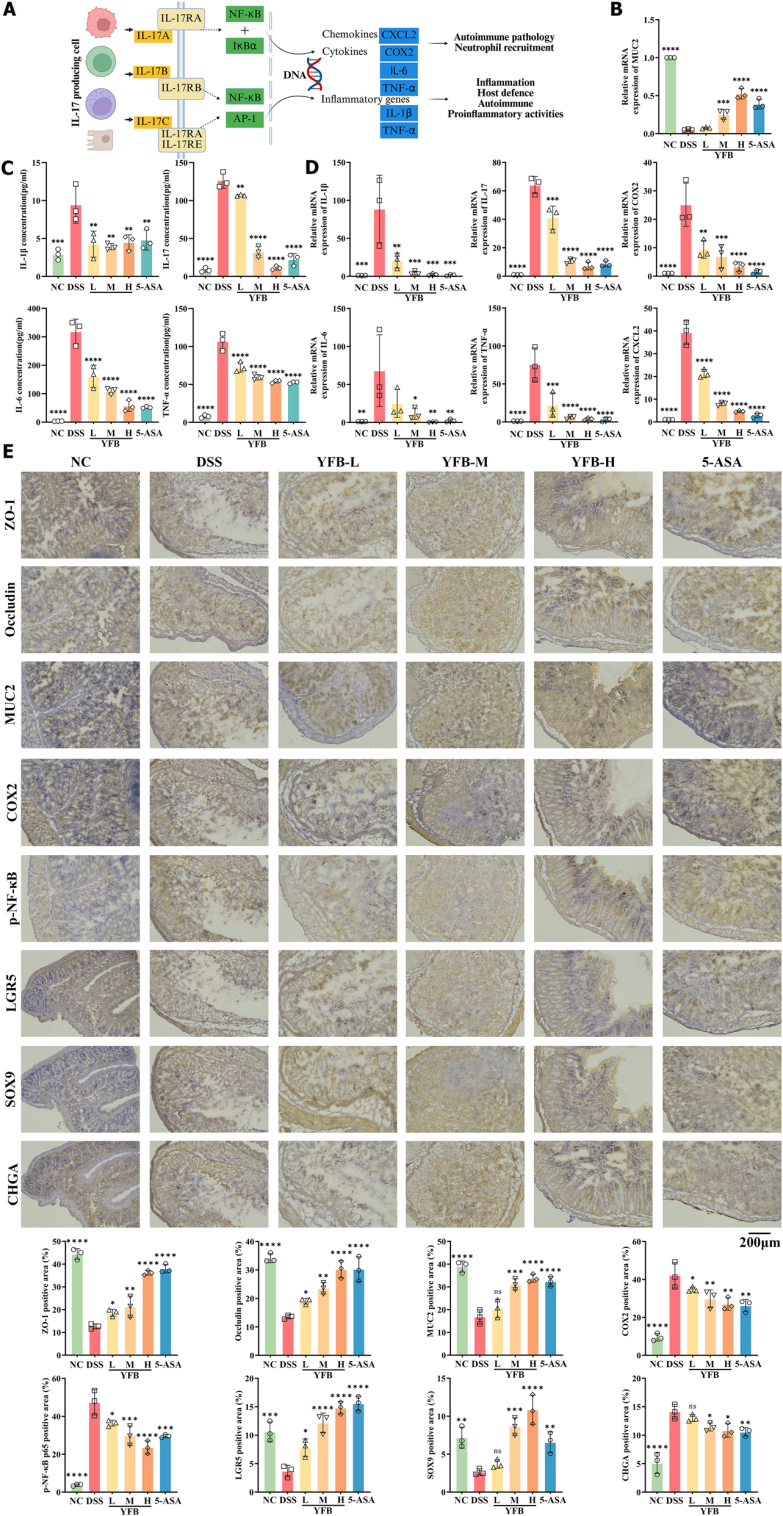
YFB attenuates inflammation, restores barrier function, and promotes epithelial repair in DSS-induced colitis. **A** Schematic diagram of the working hypothesis, illustrating the IL-17/NF-κB signaling pathway and its downstream inflammatory molecules. **B** mRNA level of *Muc2*. **C** Protein levels of inflammatory cytokines (ELISA). **D** mRNA levels of inflammatory and barrier-related genes (qPCR). **E** Representative immunohistochemistry images of indicated proteins in colon sections. Data are presented as mean ± SD (n = 5). One-way ANOVA with post hoc test was used for statistical analysis. *p* < 0.05(*), *p* < 0.01(**), *p* < 0.001(***), *p* < 0.0001(****) versus the DSS group; *NC* normal control, *DSS* DSS model, *YFB-L/M/H* YFB low/medium/high dose, *5-ASA* positive control

Network pharmacology predicted the involvement of the IL-17 pathway in YFB’s action (Fig. 1I). We therefore investigated key aspects of this and related pathways, as outlined in our working model (Fig. 4A). Consistent with the model, YFB restored intestinal barrier components compromised by colitis. The DSS-induced suppression of the mucin gene *Muc2* was reversed after YFB treatment (Fig. 4B). DSS challenge significantly elevated both mRNA and protein levels of the pro-inflammatory cytokines IL-1β, IL-6, IL-17A, and TNF-α. YFB treatment dose-dependently reduced these elevations, showing an effect comparable to 5-ASA. Similarly, the expression of inflammatory mediators *Cxcl2* and *Cox2* was upregulated by DSS and downregulated by YFB (Fig. 4C, D).

Immunohistochemical analysis confirmed the restoration of the mucus barrier, showing increased expression of MUC2 protein. Furthermore, immunohistochemistry results demonstrated that YFB upregulated the tight junction proteins ZO-1 and occludin (Fig. 4E), indicating enhancement of the physical barrier. Assessment of epithelial repair markers revealed that DSS colitis severely reduced the abundance of LGR5 + intestinal stem cells and SOX9 + progenitor cells, while increasing CHGA + enteroendocrine cells (Fig. 4E). YFB treatment effectively restored LGR5 and SOX9 expression and normalized CHGA levels (Fig. 4E). At the signaling level, YFB’s anti-inflammatory effect was associated with inhibition of the NF-κB pathway, as demonstrated by reduced nuclear accumulation of phosphorylated p65 (p-p65) and decreased COX2 expression (Fig. 4E).

### YFB treatment was associated with reversal of DSS-induced gut microbiota dysbiosis

**Fig. 5:**
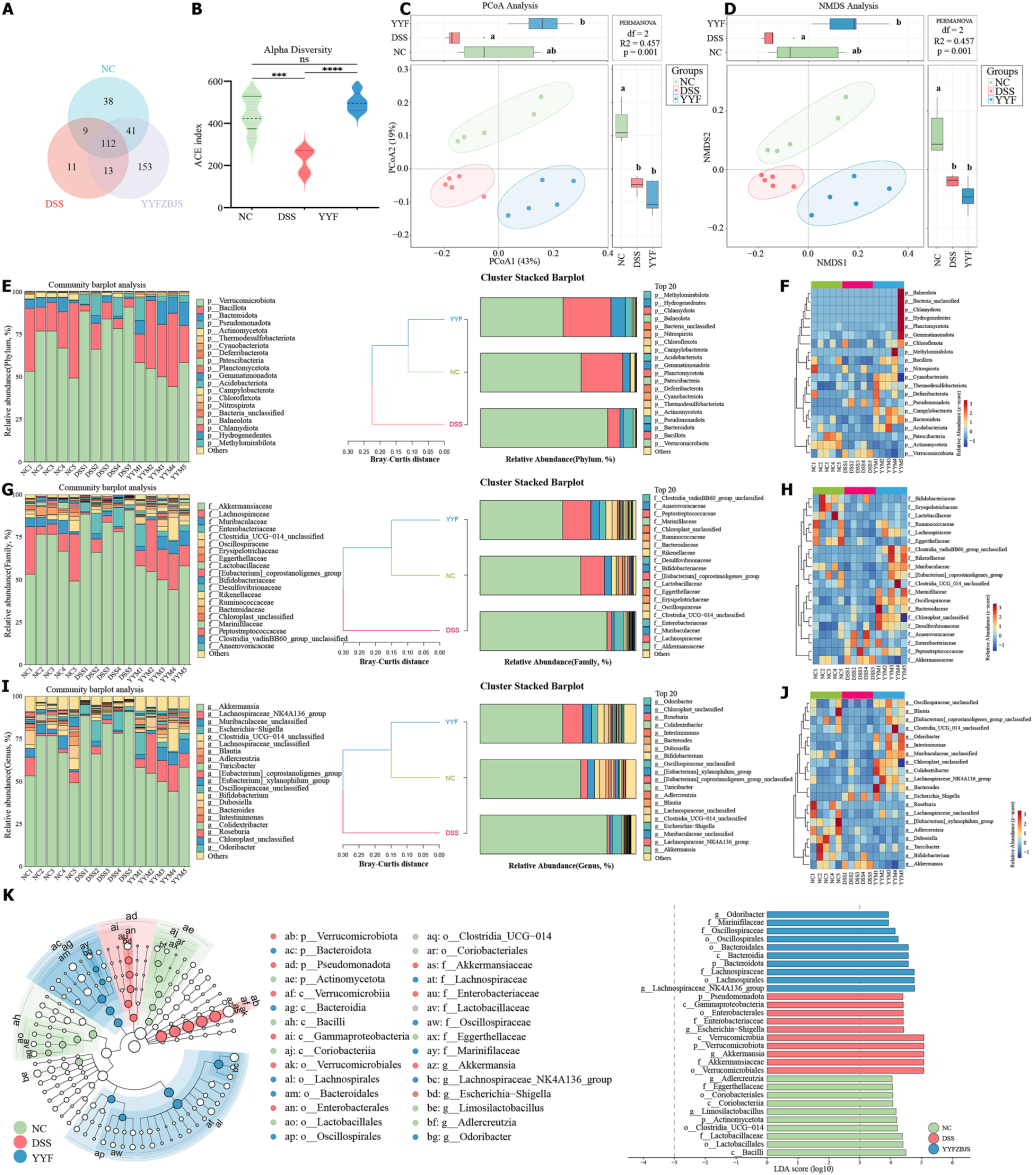
YFB treatment was associated with restoration of gut microbiota homeostasis in DSS-induced colitis. **A** Venn diagram of unique and shared ASVs. **B** Alpha diversity indices (Chao1, Shannon). **C** PCoA and **D** NMDS plots based on Bray–Curtis distance. **E**, **F** Relative abundance at the phylum level (bar plot and heatmap). **G**, **H** Relative abundance at the family level. **I**, **J** Relative abundance at the genus level. **K** Cladogram and LDA scores from LEfSe analysis (LDA > 4.0). Data in panels **B**, **E**–**J** are presented as mean ± SD (n = 5). Statistical tests are indicated in the respective panels. *p* < 0.05 (*), *p* < 0.01 (**), *p* < 0.001 (***),* p* < 0.0001 (****). *NC* normal control, *DSS* DSS model, *YFB-L/M/H* YFB low/medium/high dose

We analyzed fecal microbiota by 16S rRNA gene sequencing. Venn analysis showed distinct microbial signatures: the NC, DSS, and YFB groups harbored 200, 145, and 319 unique amplicon sequence variants (ASVs), respectively, with 112 shared core ASVs (Fig. 5A).

Alpha diversity (Chao1, Shannon indices) was significantly reduced in the DSS group, indicating loss of richness and diversity. YFB treatment restored these indices to levels comparable to the NC group (Fig. 5B). Beta diversity analysis (PCoA, NMDS) revealed a clear separation of microbial communities among groups (PERMANOVA, R^2^ = 0.457, *p* = 0.001), with the YFB group clustering closer to the NC group and away from the DSS group (Fig. 5C, D).

Compositional analysis revealed that DSS colitis decreased the relative abundance of Bacteroidota and Bacillota, but increased Verrucomicrobiota at the phylum level. YFB intervention reversed these shifts. These shifts were partially reversed in the YFB-treated group (Fig. 5E, F). At the family level, DSS reduced beneficial taxa (e.g., *Lachnospiraceae*, *Lactobacillaceae*) and promoted the expansion of *Enterobacteriaceae*. In the YFB-treated group, the abundance of beneficial taxa was increased, while Enterobacteriaceae was decreased (Fig. 5G, H). At the genus level, DSS induced a marked increase in *Akkermansia* and *Escherichia-Shigella*, and these changes were attenuated in the YFB-treated group. Conversely, the YFB-treated group showed enrichment of beneficial genera, including the short-chain fatty acid producer *Lachnospiraceae_NK4A136_group* (Fig. 5I, J).

LEfSe analysis identified group-specific biomarker taxa (LDA > 4.0) (Fig. 5K). The NC group was enriched in *Lachnospirales*/*Lachnospiraceae*; the DSS group in *Enterobacteriaceae*/*Escherichia-Shigella*; and the YFB group in *Verrucomicrobiota* and its derived taxa (e.g., *Akkermansiaceae*, *Akkermansia*). Notably, although YFB reduced the DSS-driven overgrowth of *Akkermansia*, it maintained its presence as a signature taxon, suggesting a role in reconstructing a mucosa-associated microbial community that inhibits harmful bacteria.

### YFB treatment was associated with alterations in the fecal metabolomic profile

**Fig. 6:**
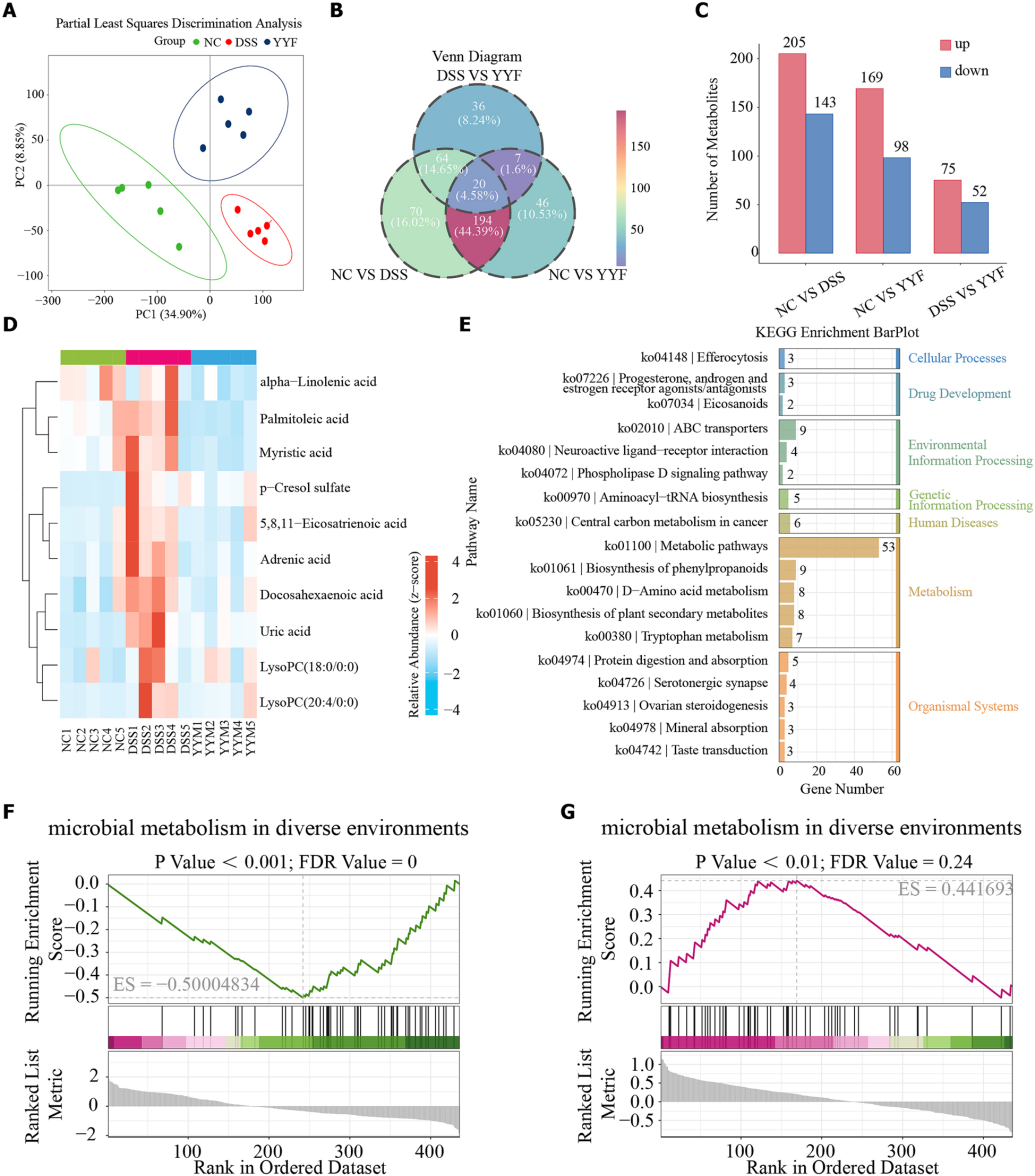
YFB treatment was associated with alterations in the fecal metabolome in DSS-induced colitis. **A** PLS-DA score plot. **B** Venn diagram of differentially abundant metabolites (DAMs) between comparison groups. **C** Number of up- and down-regulated DAMs in each comparison. **D** Heatmap of hierarchical clustering of key DAMs. **E** KEGG pathway enrichment analysis of DAMs. **F**, **G** Gene set enrichment analysis (GSEA) plots for the “microbial metabolism in diverse environments” pathway in NC vs. DSS (**F**) and DSS vs. YFB (**G**) comparisons. *NES* normalized enrichment score, *FDR* false discovery rate

To determine if microbiota changes were accompanied by metabolic shifts, we performed untargeted metabolomics on fecal samples. PLS-DA showed clear separation between the DSS and YFB groups (Fig. 6A). We identified 194, 205, and 143 differentially abundant metabolites in DSS vs. NC, NC vs. YFB, and DSS vs. YFB comparisons, respectively, with 36 common to all comparisons (Fig. 6B). Overall, DSS treatment broadly elevated metabolite levels, while YFB treatment tended to restore them toward NC levels (Fig. 6C).

Cluster heatmap analysis of key differential metabolites confirmed extensive metabolic disturbances induced by DSS. Specifically, alterations were observed in fatty acids [e.g., palmitoleic acid (*p* = 0.0279), myristic acid (*p* = 0.0108), α-linolenic acid (*p* = 0.0207)], and other critical metabolites including uric acid (*p* = 0.0005), and several lysophosphatidylcholines (LysoPCs), all identified at MSI confidence Level 2. Notably, YFB treatment effectively reversed the dysregulated levels of most of these metabolites, restoring their expression patterns to near-normal conditions (Fig. 6D).

KEGG pathway enrichment analysis showed that differential metabolites were significantly associated with pathways including tryptophan metabolism, ABC transporters, and efferocytosis (Fig. 6E). Gene set enrichment analysis (GSEA) further indicated that the “microbial metabolism in diverse environments” pathway was markedly activated in the DSS group (vs. NC, *p* < 0.001), and this activation was significantly suppressed by YFB treatment (vs. DSS, *p* < 0.01) (Fig. 6F, G).

## Discussion

Prior studies have established the therapeutic efficacy of Yiyi Fuzi Baijiang formula (YFB) against colitis. Yang [35–37] demonstrated that YFB repairs the intestinal epithelial barrier in rats by inhibiting the TLR4/NF-κB/NLRP3 signaling pathway. Zhang [40] employed network pharmacology and 16S rRNA sequencing to report the modulatory effects of YFB on gut microbiota composition. Building on these findings, the present study offers several incremental contributions. First, using untargeted metabolomics, we found that YFB treatment was associated with changes in the metabolic function of the gut microbiota, extending beyond a mere alteration of its compositional profile. Second, our data suggest that YFB treatment was associated with restoration of LGR5-positive intestinal stem cells and SOX9-positive progenitor cells, while concurrently normalizing the aberrant expansion of CHGA-positive enteroendocrine cells—a mechanistic aspect of YFB that has not been previously documented. Third, we employed qPCR, ELISA, and immunohistochemistry to validate the core pathways (NF-κB, IL-17, and TNF) predicted by network pharmacology; notably, our NF-κB data corroborate the findings reported by Yang et al. in rats.

This study systematically investigated the protective mechanisms of Yiyi Fuzi Baijiang formula (YFB) against DSS-induced acute colitis by integrating network pharmacology, phytochemical analysis, in vivo validation, 16S rRNA gene sequencing, and untargeted metabolomics. Our results demonstrate that when administered concomitantly with DSS, YFB protects against colitis through a multi-targeted synergy, effectively modulating the interconnected gut barrier–microbiota–metabolism axis.

Network pharmacology provided a strategic starting point, predicting that flavonoids like quercetin and kaempferol in YFB might act via the IL-17 and related pathways [4]. Subsequent UPLC-Q-TOF-MS/MS analysis confirmed the presence of these and other flavonoids, and importantly, identified structurally optimized forms such as acylated and methylated glycosides. Such natural modifications are known to enhance bioavailability and stability [17, 29, 39], offering a plausible phytochemical basis for YFB’s in vivo efficacy. It should be noted that the predicted oral bioavailability of flavonoid glycosides in Table 3 is relatively low, but this does not diminish their in vivo efficacy. This is because flavonoid glycosides can be deglycosylated by gut microbiota in the intestine to release their aglycones (e.g., quercetin, kaempferol), which are the actual bioactive forms. In addition, due to the limited coverage of public databases, OB/DL values were not available for all identified glycosides, which we acknowledge as a limitation. To compensate for this limitation, targeted quantitative MS analysis was performed on several major components with relatively high abundance in YFB.

The in vivo efficacy of YFB was evident. It ameliorated disease activity, colon shortening, and histopathological damage. A key mechanism was the restoration of intestinal barrier integrity, evidenced by upregulated expression of tight junction proteins (ZO-1, occludin) and mucin (MUC2). Barrier function is tightly regulated by local inflammation [19] and the microbiota [25]. DSS challenge triggered a pro-inflammatory cytokine storm (TNF-α, IL-6, IL-17A) and NF-κB hyperactivation, all of which were suppressed by YFB. This anti-inflammatory action, potentially driven by components like quercetin, likely creates a microenvironment conducive to barrier repair. Concurrently, DSS-induced dysbiosis (e.g., *Enterobacteriaceae* bloom) can disrupt tight junctions and fuel inflammation. YFB’s correction of this dysbiosis may thus break this vicious cycle, facilitating barrier recovery. The medium dose of YFB (6.65 g/kg) was selected as the human equivalent dose based on the prescribed dosage recorded in the classic formula, converted according to body surface area. The median values of some parameters in the high dose group were better than those in the medium dose group. However, given that Aconite (Fuzi) is classified as a toxic herb by the Chinese Pharmacopoeia with a defined clinical dosage range, we maintained rigor and did not perform further statistical tests. The lack of significantly increased efficacy in some high-dose parameters may be related to safety considerations and species differences. Taken together, YFB may exhibit a bell-shaped dose–response curve.

Beyond barrier repair, YFB profoundly influenced epithelial regeneration. IHC analysis revealed that DSS colitis depleted LGR5 + intestinal stem cells and SOX9 + progenitor cells—cell populations essential for epithelial self-renewal and differentiation. Simultaneously, it increased CHGA + enteroendocrine cells (EECs), a population that includes serotonin-producing enterochromaffin cells. Excessive serotonin signaling is implicated in exacerbating intestinal inflammation [1]. YFB treatment restored LGR5 and SOX9 expression and normalized CHGA levels, indicating its capacity to rebalance epithelial differentiation and promote regeneration, which synergistically strengthens the mucosal barrier.

YFB treatment was associated with systematic alterations in the gut ecosystem. These alterations included reversal of DSS-induced dysbiosis, reduced overgrowth of Escherichia–Shigella, and modulated Akkermansia levels. While Akkermansia is generally recognized as a beneficial gut commensal, its role in DSS-induced colitis is multifaceted. Recent studies indicate that during intestinal inflammation, fast-replicating taxa such as Akkermansia may acquire a growth advantage [2], and different strains of Akkermansia muciniphila exhibit variable effects on colitis [22]. Therefore, the increased abundance of Akkermansia in the DSS group likely reflects a complex stress response, and its reduction by YFB suggests restoration of microbial homeostasis. Notably, the YFB-treated group showed enrichment of beneficial SCFA-producing bacteria such as *Lachnospiraceae_NK4A136_group*. This microbial restructuring was accompanied by a correction of the distorted fecal metabolome. YFB normalized levels of dysregulated fatty acids (e.g., palmitoleic, α-linolenic acid) and suppressed the aberrant activation of the “microbial metabolism in diverse environments” pathway (GSEA). In addition, KEGG pathway enrichment analysis showed that YFB significantly modulated the tryptophan metabolism pathway (ko00380). Tryptophan metabolites play important roles in maintaining intestinal immune homeostasis, and their dysregulation is closely associated with IBD pathogenesis. This result suggests that modulation of tryptophan metabolism may contribute to the protective effects of YFB. SCFAs and other microbially modulated metabolites are crucial for maintaining barrier integrity and immune homeostasis [18, 26]. Thus, YFB treatment appears to be associated with alterations in both microbial composition and its collective metabolic function, which may generate beneficial signals that potentially support barrier function and dampen inflammation.

**Fig. 7:**
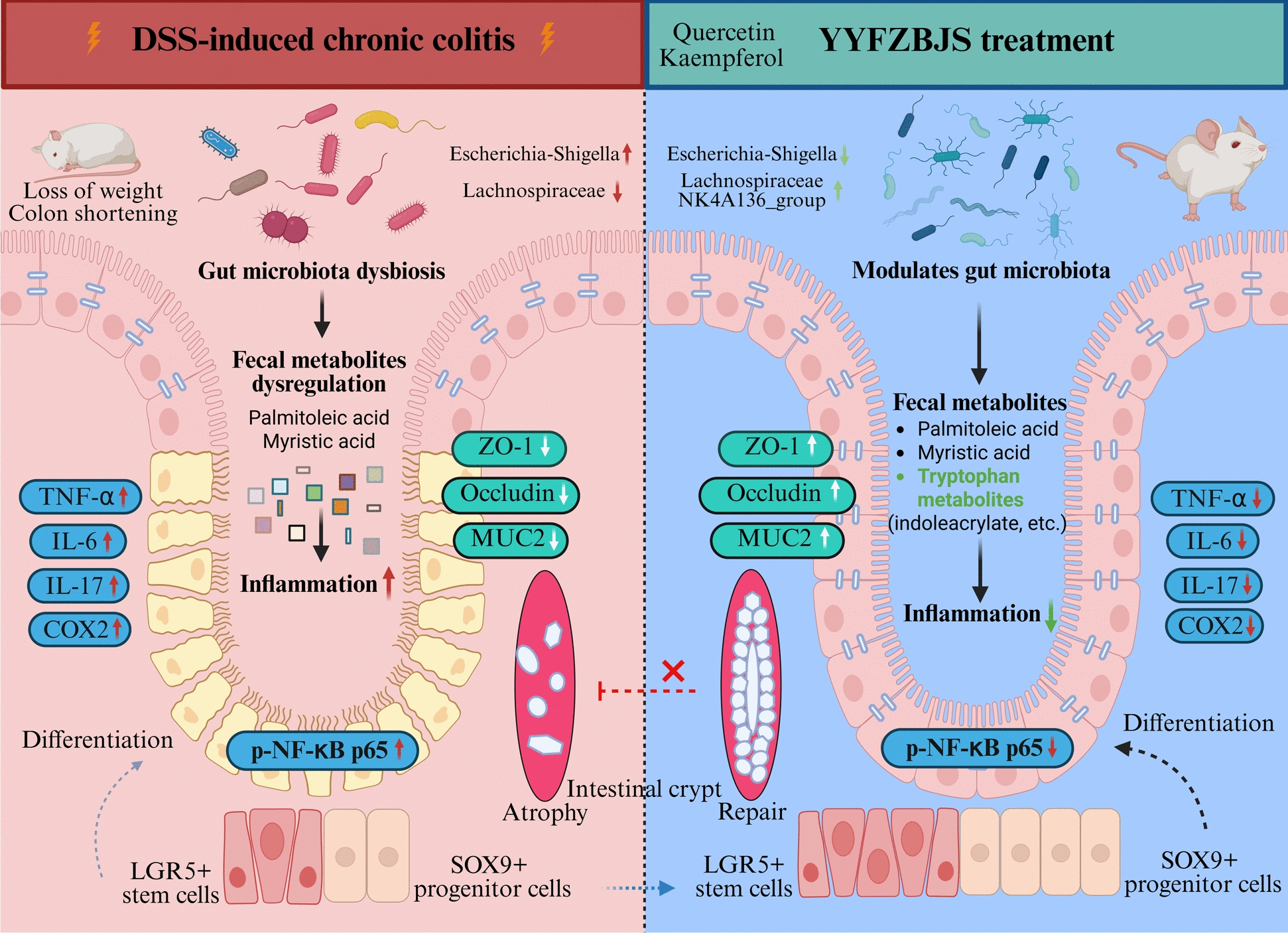
Schematic illustration of the multi-target mechanisms by which Yiyi Fuzi Baijiang formula (YFB) protects against DSS-induced colitis through modulation of the gut barrier–microbiota–metabolism axis

To comprehensively summarize the multi-target mechanisms of YFB against colitis, we propose an integrated schematic diagram (Fig. 7). This model illustrates that YFB, through its bioactive components (e.g., quercetin and kaempferol), orchestrates a systemic restoration of the gut barrier–microbiota–metabolism axis. Specifically, YFB inhibits NF-κB-driven inflammation, upregulates tight junction proteins (ZO-1, occludin) and mucin (MUC2), promotes the regeneration of intestinal stem/progenitor cells (LGR5⁺/SOX9⁺), and normalizes enteroendocrine cell (CHGA⁺) populations. Concurrently, YFB reshapes gut microbiota composition, enriches beneficial bacteria (e.g., *Lachnospiraceae*), suppresses opportunistic pathogens, and corrects dysregulated fecal metabolites. These coordinated actions collectively contribute to the alleviation of DSS-induced colitis, highlighting the holistic protective potential of YFB.

In conclusion, YFB protects against DSS-induced colitis through a multi-component, multi-pathway network. Its active constituents likely inhibit pivotal inflammatory pathways like NF-κB. Simultaneously, they are associated with alterations in the gut microbiota and its metabolic output, which may contribute to barrier integrity and suppression of inflammation. These actions collectively restore homeostasis across the gut barrier–microbiota–metabolism axis. Our findings provide a scientific rationale for the clinical application of YFB and exemplify the holistic, system-regulating potential of TCM formulations in treating complex multi-factorial diseases like IBD. Considering the multi-target effects of YFB on inflammation, barrier repair, and microbiota modulation, along with its protective effects observed in the acute colitis model in this study, YFB may have potential value in preventing acute flares of chronic intestinal inflammation. Future preclinical and clinical studies are warranted to explore this possibility. Given its multi-targeted actions on inflammation, barrier repair, and microbiota modulation, YFB may also represent a promising candidate for managing immune-related adverse events such as ICI-associated colitis, warranting further investigation in relevant preclinical and clinical settings.

Network pharmacology was used for hypothesis generation, and the predicted pathways are common. Direct binding of YFB components to the targets was not validated, which is a limitation of this study. Several limitations should be acknowledged. The sample size of five mice per group is relatively small for microbiome and metabolomics analyses and may affect statistical power. In addition, our microbiota data demonstrate correlations rather than causation, and fecal microbiota transplantation (FMT) experiments are required to determine causality. Furthermore, only male mice were used in this study, and given the sex-specific differences reported in IBD, future studies should include female mice to evaluate potential sex-dependent responses to YFB. Also, the concomitant administration design used in this study evaluates protective rather than therapeutic effects; a therapeutic protocol (treatment after colitis establishment) would better mimic clinical practice and will be pursued in future studies. Future studies with larger cohorts, FMT, and inclusion of both sexes, as well as further investigation of key molecular pathways, are needed to elucidate the mechanisms of YFB.

## Conclusion

This study demonstrates that Yiyi Fuzi Baijiang formula (YFB) prevents DSS-induced acute colitis in mice through synergistic, multi-target mechanisms. YFB’s protective effect involves a systematic regulatory process: it directly inhibits NF-κB activation and pro-inflammatory cytokine production, while concurrently being associated with alterations in the gut microbiota, including suppression of opportunistic pathogens and enrichment of beneficial bacteria that are known to produce short-chain fatty acids. These actions collectively restore intestinal barrier integrity and correct host–microbial co-metabolic disturbances. Ultimately, YFB exerts its efficacy via coordinated regulation of the gut barrier–microbiota–metabolism axis. Our findings provide a robust scientific basis for the clinical application of YFB and underscore the value of traditional Chinese medicine formulations in treating complex, multifactorial diseases through system-level regulation. In addition, SCFA levels were not directly measured in this study; the mention of SCFAs is based on the abundance changes of SCFA-producing bacteria. Targeted metabolomics is required for definitive quantification of SCFAs in future studies.

## Supplementary Information

Supplementary material 1.

Supplementary material 2.

## Data Availability

No datasets were generated or analysed during the current study.

## Abbreviations

ASV: Amplicon sequence variant
CHGA: Chromogranin A
COX2: Cyclooxygenase-2
DAI: Disease activity index
DSS: Dextran sulfate sodium
ELISA: Enzyme-linked immunosorbent assay
GSEA: Gene set enrichment analysis
GO: Gene ontology
H: High
IBD: Inflammatory bowel disease
IHC: Immunohistochemistry
IL-1β/6/17A: Interleukin-1 Beta/6/17A
KEGG: Kyoto Encyclopedia of Genes and Genomes
L: Low
LEfSe: Linear discriminant analysis effect size
LGR5: Leucine-rich repeat-containing G-protein coupled receptor 5
M: Medium
MUC2: Mucin 2
NF-κB: Nuclear factor kappa-light-chain-enhancer of activated B cells
NMDS: Non-metric multidimensional scaling
PCoA: Principal co-ordinates analysis
PLS-DA: Partial least squares-discriminant analysis
PPI: Protein–protein interaction
RT-qPCR: Quantitative real-time polymerase chain reaction
SCFA: Short-chain fatty acid
SOX9: SRY-box transcription factor 9
TCM: Traditional Chinese Medicine
TNF-α: Tumor necrosis factor-alpha
UPLC-Q-TOF-MS/MS: Ultra-performance liquid chromatography quadrupole time-of-flight tandem mass spectrometry
YFB: Yiyi Fuzi Baijiang formula
ZO-1: Zonula occludens-1
5-ASA: 5-Aminosalicylic acid
