# Supplementary material for: Yiyi Fuzi Baijiang formula protects against DSS-induced colitis by orchestrating the gut barrier-microbiota-metabolism axis
Source: Chin Med. 2026 Jul 23;21:202. doi: 10.1186/s13020-026-01478-x (PMC13393498; doi:10.1186/s13020-026-01478-x)

1. Quercetin

1.1. Quercetin 3-(2Gal-apiosylrobinobioside)：


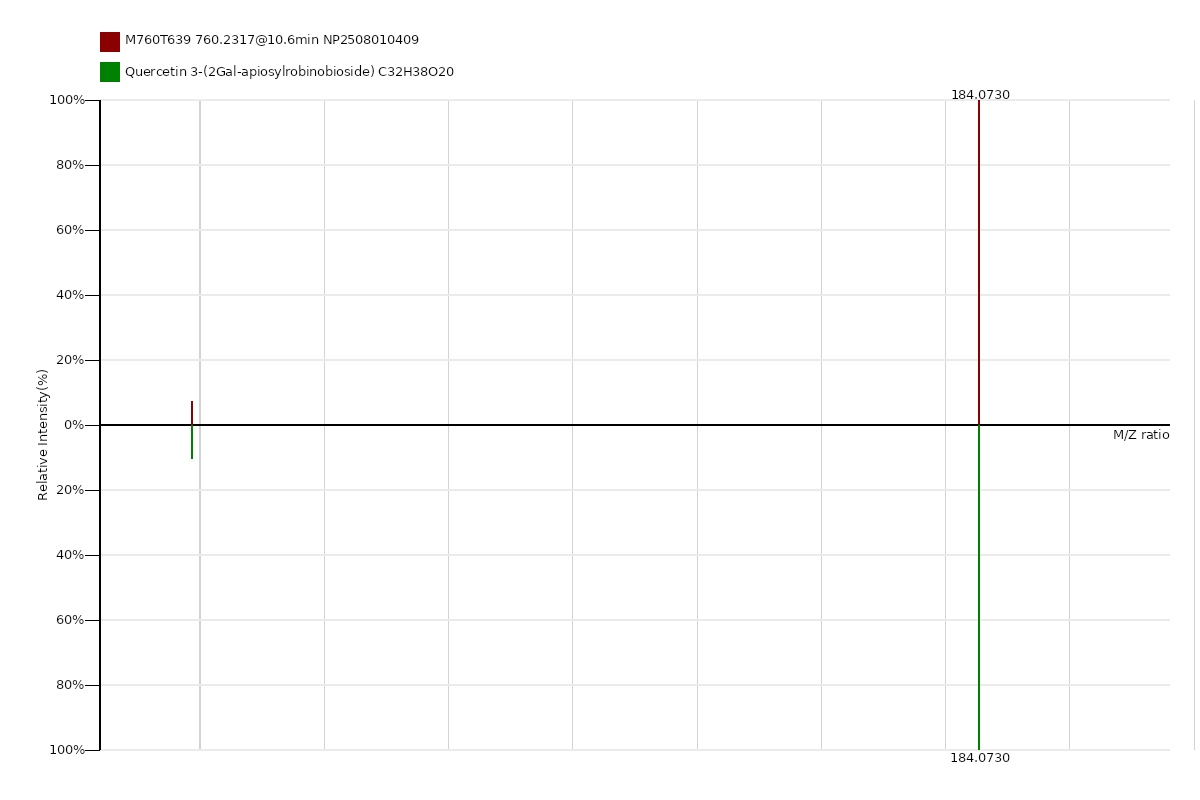


1.2. Quercetin 3-(3',6'-di-p-coumarylglucoside)


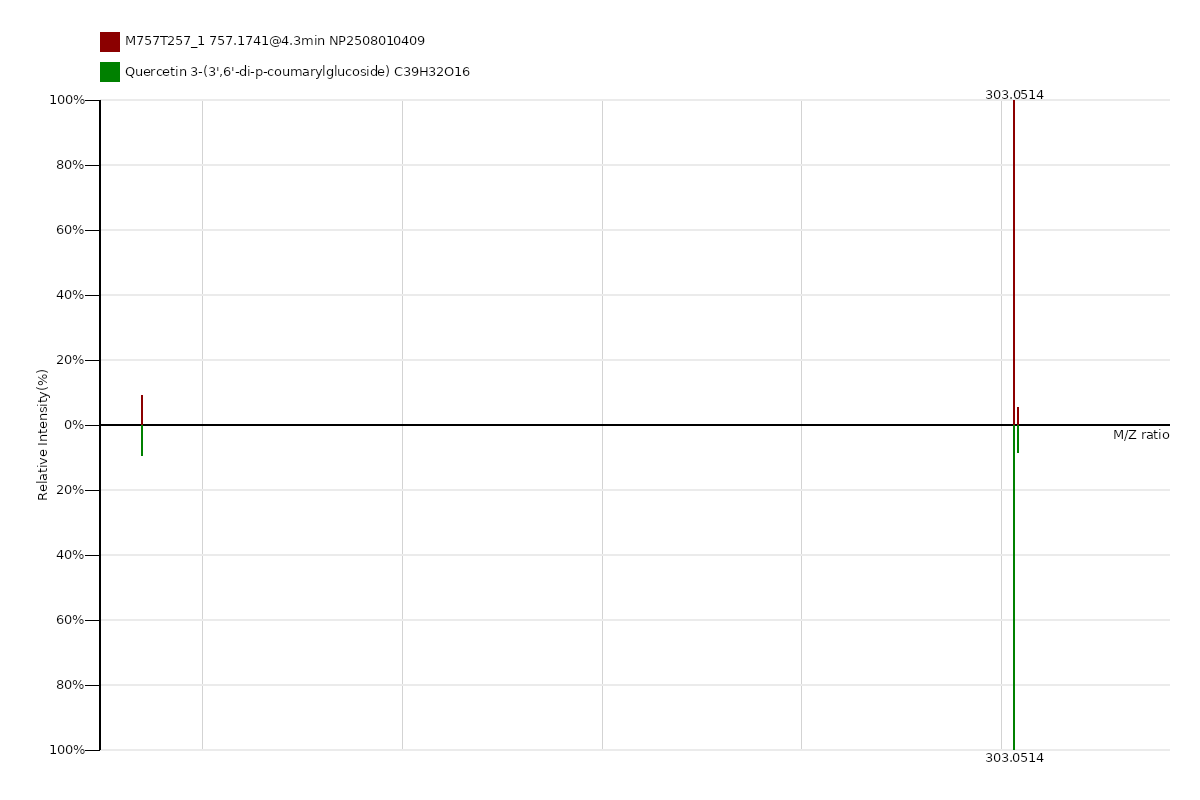


1.3. Quercetin 3-galactoside 7-rhamnoside


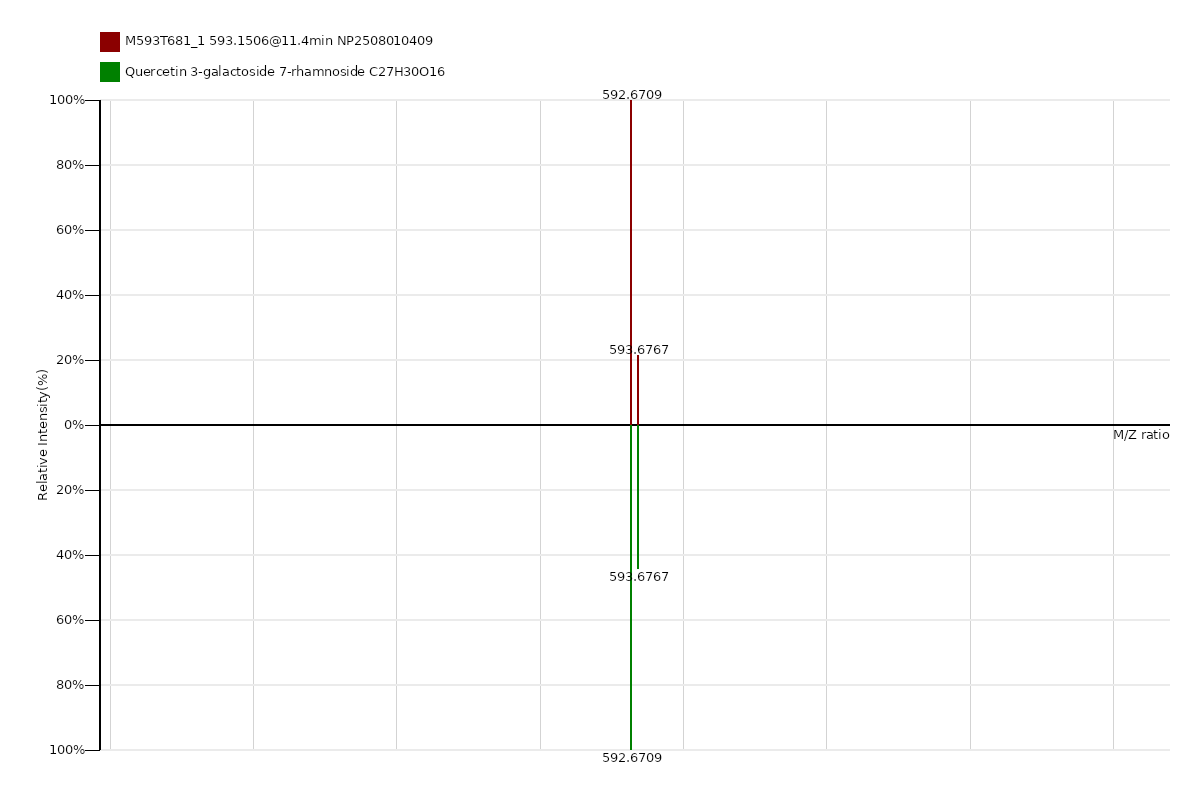


1.4. Quercetin 3-neohesperidoside-7-rhamnoside


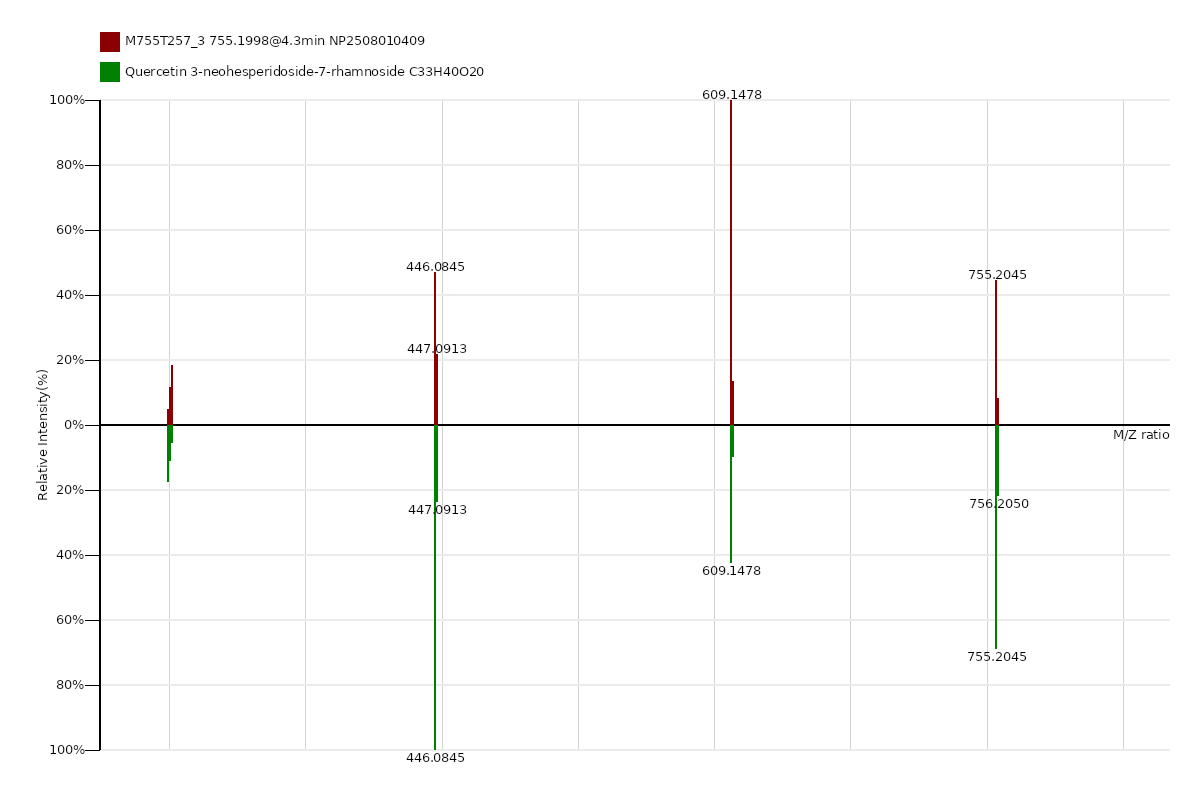


1.5. Quercetin 7,3,4-trimethyl ether 3-alpha-L-arabinopyranoside


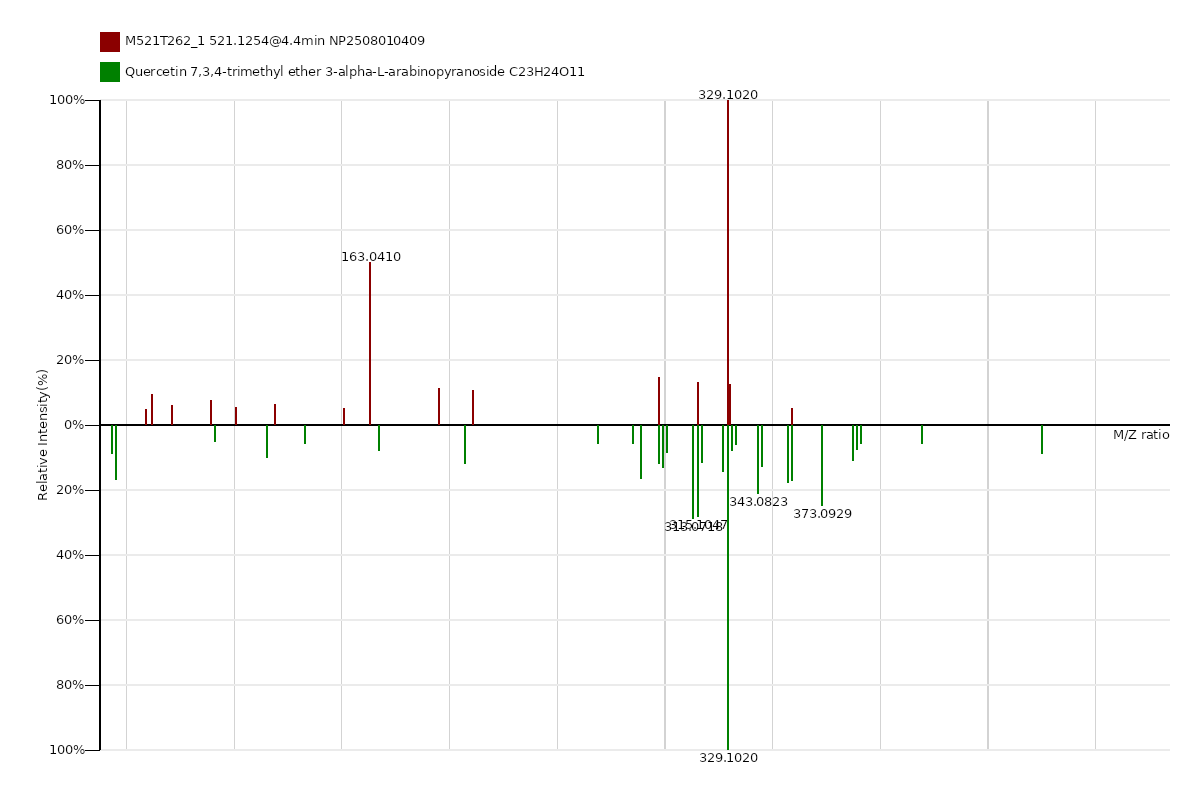


2. Kaempferol

2.1 3-O-Methylkaempferol


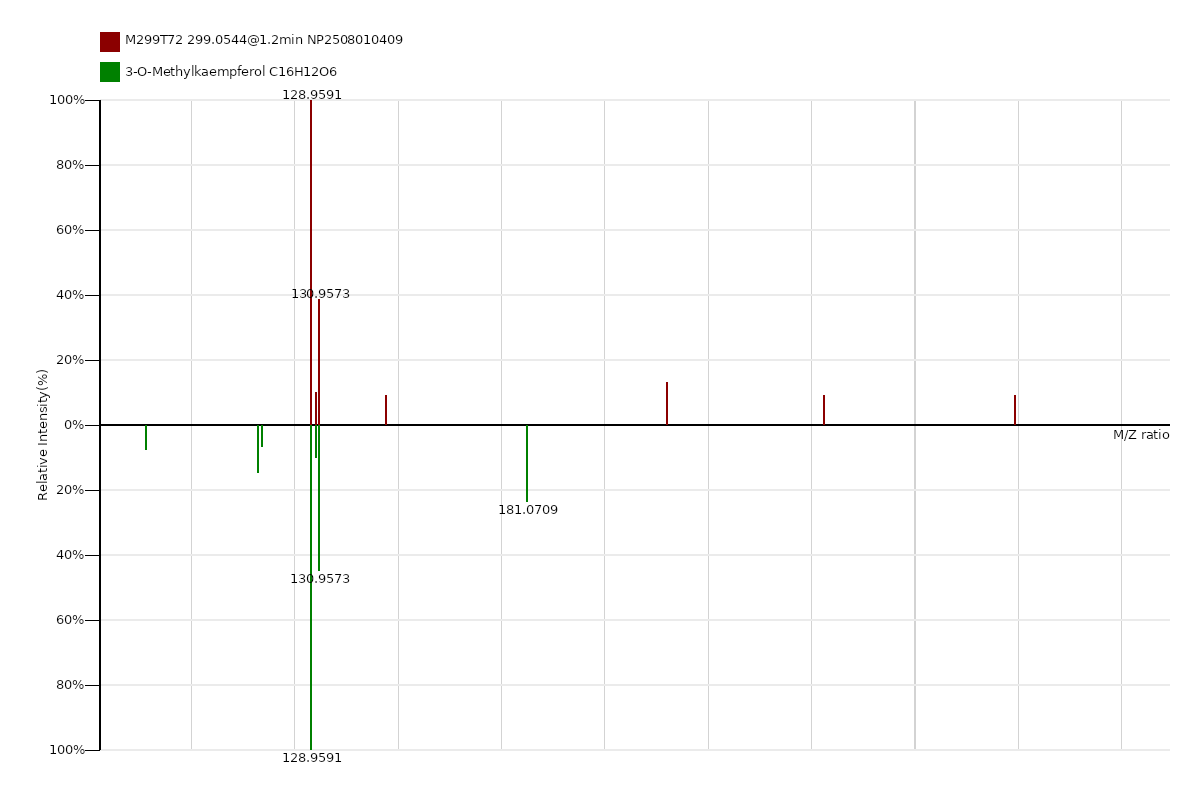


2.2 Kaempferol 3-(2',6'-di-(E)-p-coumarylglucoside)


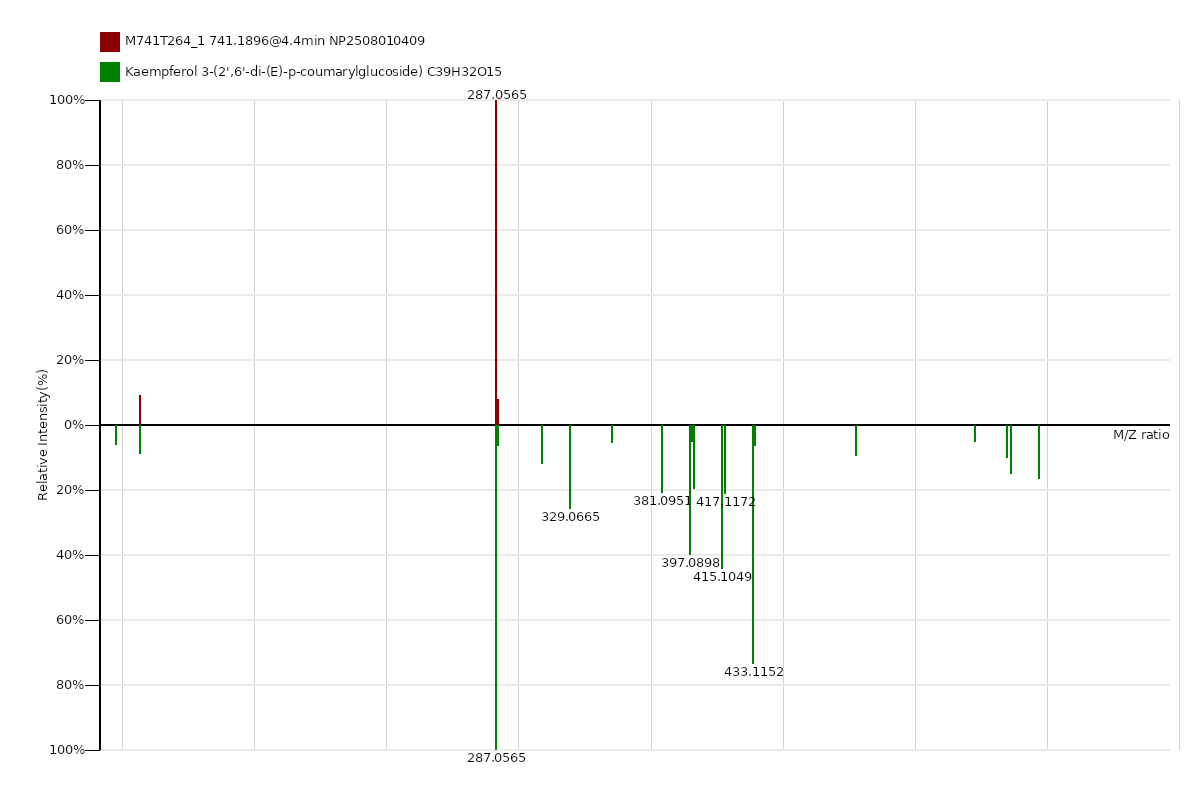


2.3 Kaempferol 3-(6'-rhamnosylsophoroside)


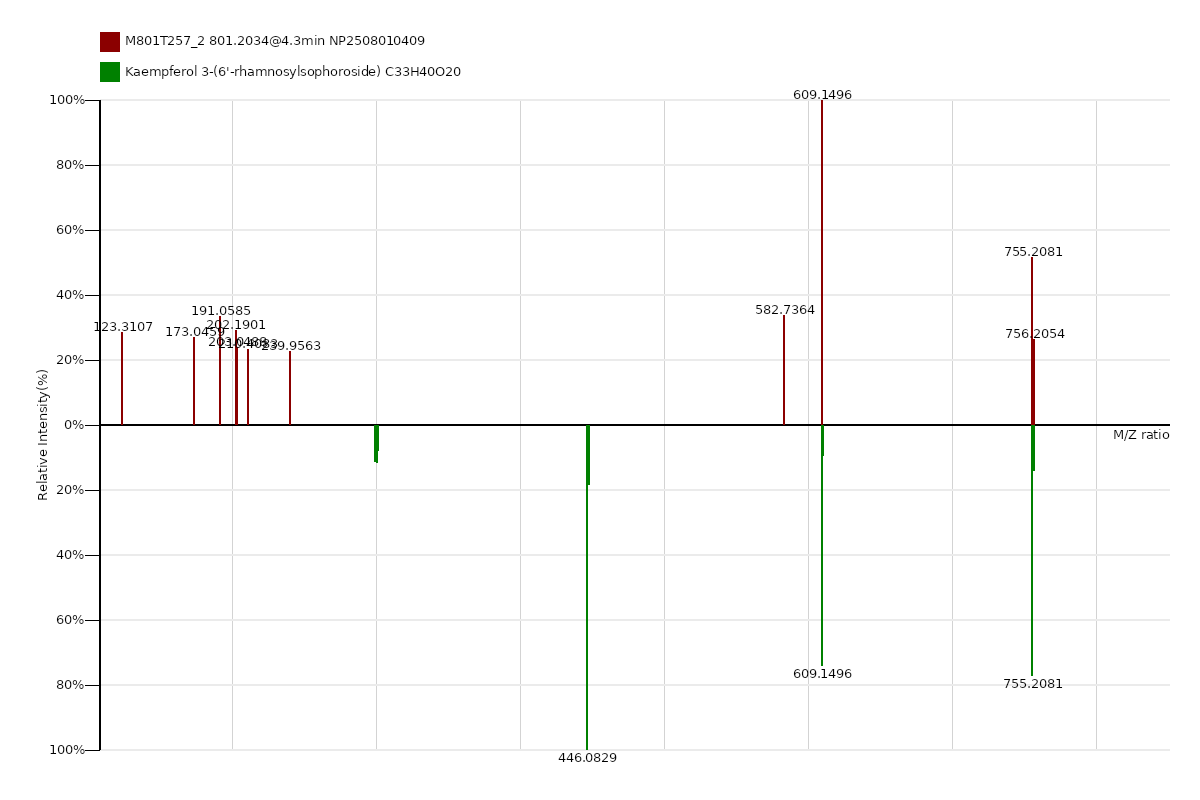


2.4 Kaempferol 3-[glucosyl-(1->3)-rhamnosyl-(1->2)-[rhamnosyl-(1->6)-galactoside]]


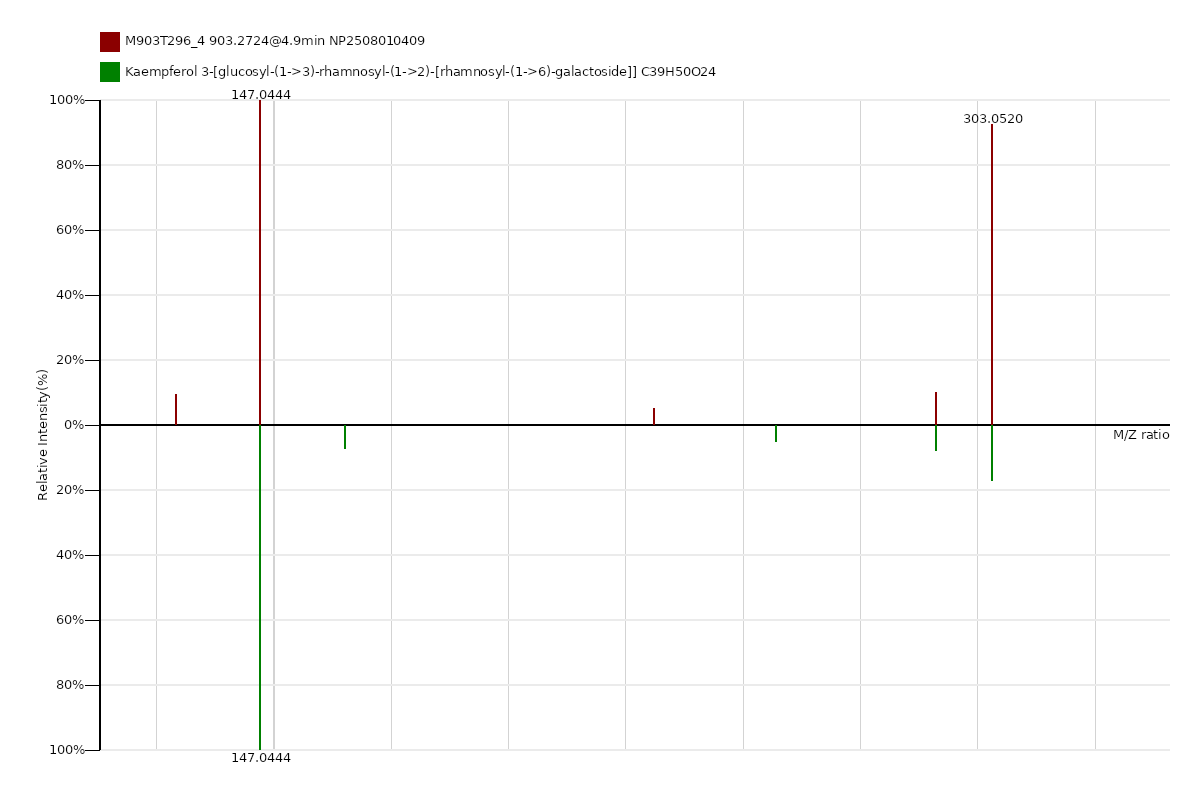


2.5 Kaempferol 3-isorhamninoside-7-rhamnoside


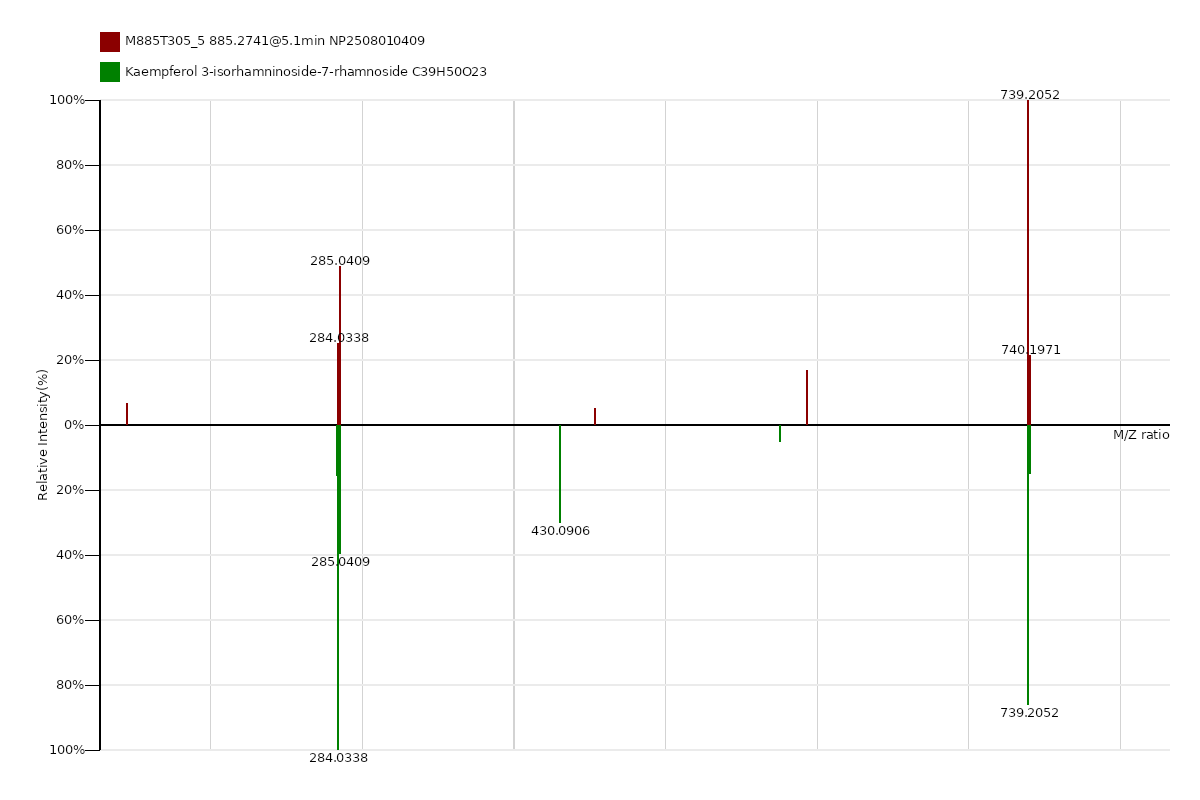


2.6 Kaempferol (aglycone)


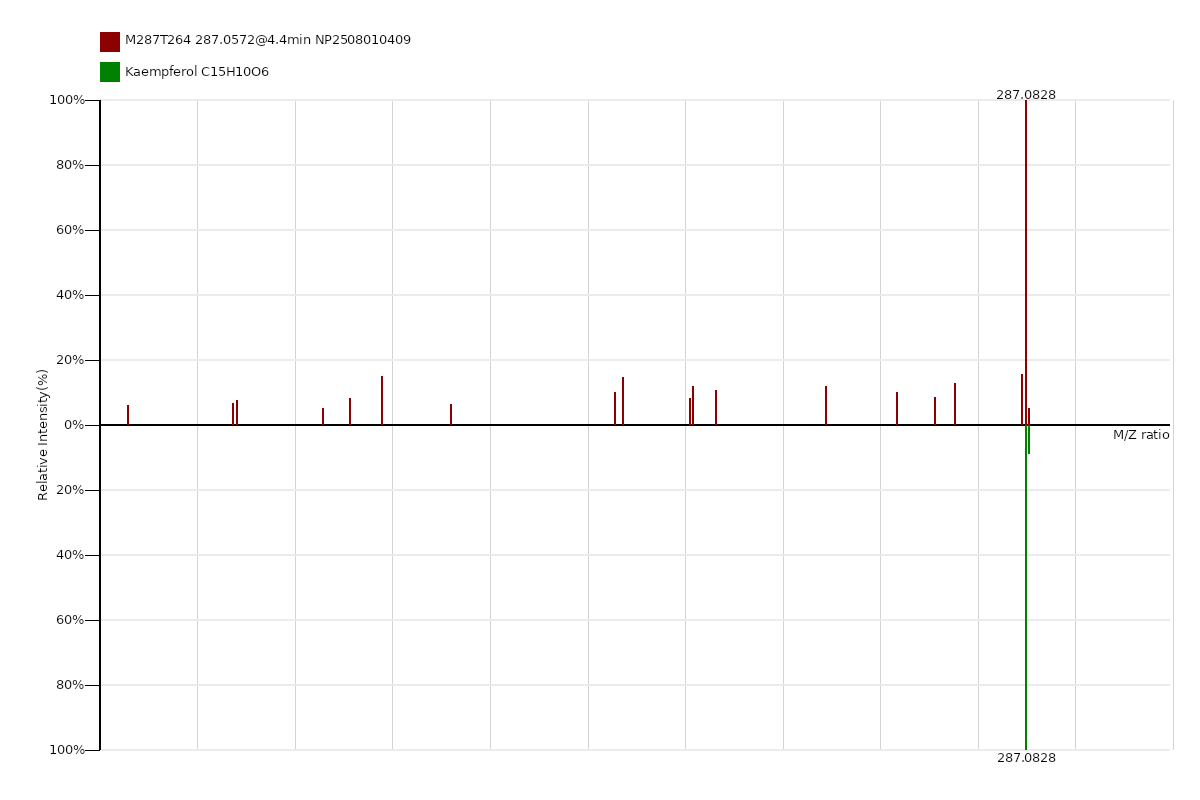


3 Acacetin 7-(6-acetylglucoside)


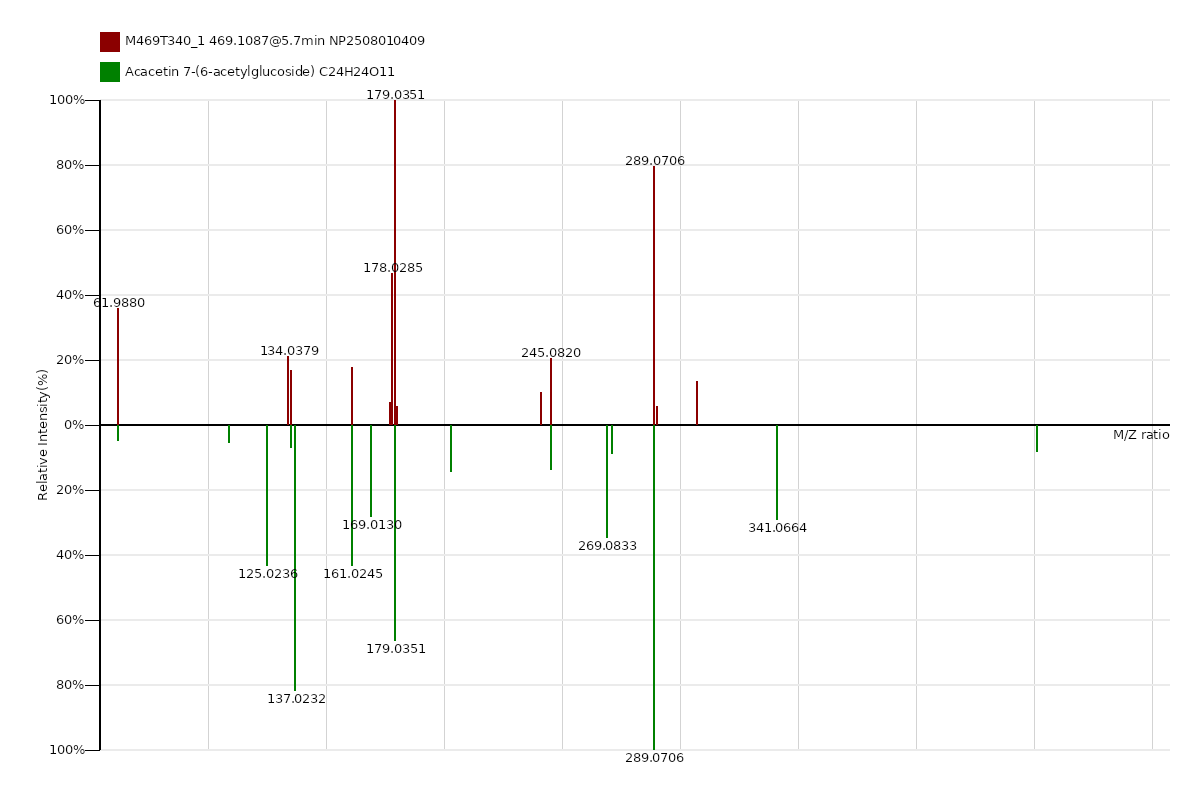


4 Isovitexin 2'-(6'-p-coumaroylglucoside)


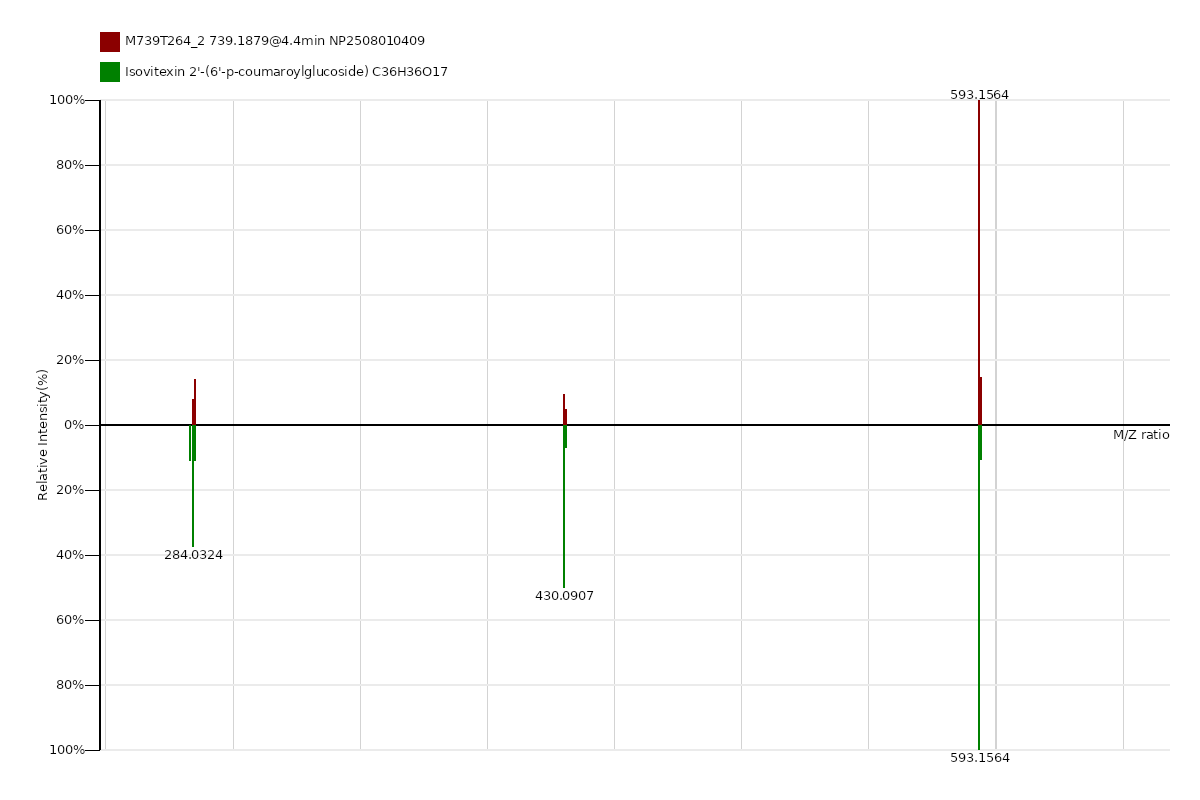

Supplement: Supplementary file 1 — Supplementary material 1. [file 13020_2026_1478_MOESM1_ESM.docx]
