# Supplementary material for: Yiyi Fuzi Baijiang formula protects against DSS-induced colitis by orchestrating the gut barrier-microbiota-metabolism axis
Source: Chin Med. 2026 Jul 23;21:202. doi: 10.1186/s13020-026-01478-x (PMC13393498; doi:10.1186/s13020-026-01478-x)
Supplement: Supplementary file 2 — Supplementary material 2. [file 13020_2026_1478_MOESM2_ESM.docx]

| **Supplementary Table S1. PPI network information of the 155 overlapping targets.** | |
| --- | --- |
| Node | Edge |
| TYR | 1 |
| SLC6A4 | 1 |
| SLC2A4 | 1 |
| SCN5A | 1 |
| PPARD | 1 |
| PPARA | 1 |
| PLAU | 1 |
| ODC1 | 1 |
| MPO | 1 |
| LTA4H | 1 |
| FASN | 1 |
| DUOX2 | 1 |
| DRD1 | 1 |
| DPP4 | 1 |
| COL3A1 | 1 |
| CHRM3 | 1 |
| CHRM1 | 1 |
| ADRB2 | 1 |
| ADRB1 | 1 |
| ACACA | 1 |
| TOP2A | 2 |
| SELE | 2 |
| RASA1 | 2 |
| PTGES | 2 |
| PPP3CA | 2 |
| POR | 2 |
| NR1I2 | 2 |
| KCNH2 | 2 |
| IGF2 | 2 |
| F7 | 2 |
| CTSD | 2 |
| COL1A1 | 2 |
| APP | 2 |
| ADH1C | 2 |
| ACHE | 2 |
| THBD | 3 |
| SOD1 | 3 |
| SLC6A3 | 3 |
| PTGS1 | 3 |
| PLAT | 3 |
| NOS2 | 3 |
| MMP3 | 3 |
| MMP2 | 3 |
| MMP1 | 3 |
| MET | 3 |
| KDR | 3 |
| IRF1 | 3 |
| INSR | 3 |
| HMOX1 | 3 |
| GJA1 | 3 |
| FASLG | 3 |
| CXCL11 | 3 |
| CHEK2 | 3 |
| CHEK1 | 3 |
| CD40LG | 3 |
| ALOX5 | 3 |
| AHR | 3 |
| SPP1 | 4 |
| SERPINE1 | 4 |
| RB1 | 4 |
| PRKCB | 4 |
| PPARG | 4 |
| NQO1 | 4 |
| NOS3 | 4 |
| MCL1 | 4 |
| MAOB | 4 |
| MAOA | 4 |
| IL2RA | 4 |
| IGFBP3 | 4 |
| HSPB1 | 4 |
| HSPA5 | 4 |
| F3 | 4 |
| CYP1B1 | 4 |
| CYP19A1 | 4 |
| BIRC5 | 4 |
| BAX | 4 |
| RXRA | 5 |
| PTGS2 | 5 |
| PRKCA | 5 |
| PGR | 5 |
| PARP1 | 5 |
| NFE2L2 | 5 |
| GSTP1 | 5 |
| GSTM1 | 5 |
| GSK3B | 5 |
| ESR2 | 5 |
| ERBB3 | 5 |
| ERBB2 | 5 |
| CYP1A2 | 5 |
| AR | 5 |
| VCAM1 | 6 |
| RUNX2 | 6 |
| PCNA | 6 |
| NFKBIA | 6 |
| CAV1 | 6 |
| CASP7 | 6 |
| RAF1 | 7 |
| IKBKB | 7 |
| ICAM1 | 7 |
| EGF | 7 |
| CYP1A1 | 7 |
| CXCL2 | 7 |
| CDK1 | 7 |
| CASP8 | 7 |
| BCL2L1 | 7 |
| XIAP | 8 |
| STAT1 | 8 |
| NCF1 | 8 |
| IFNG | 8 |
| CXCL10 | 8 |
| CHUK | 8 |
| CDKN1A | 8 |
| MMP9 | 9 |
| MAPK8 | 9 |
| MAPK14 | 9 |
| EGFR | 9 |
| E2F1 | 9 |
| CYP3A4 | 9 |
| CDK4 | 9 |
| CDK2 | 9 |
| CCL2 | 9 |
| MYC | 10 |
| MDM2 | 10 |
| IL4 | 10 |
| IL1A | 10 |
| IL10 | 10 |
| HIF1A | 10 |
| CXCL8 | 10 |
| CASP9 | 10 |
| RELA | 11 |
| FOS | 11 |
| IL1B | 12 |
| CCND1 | 12 |
| MAPK1 | 13 |
| HSP90AA1 | 13 |
| BCL2 | 13 |
| CASP3 | 14 |
| TNF | 15 |
| IL6 | 15 |
| ESR1 | 15 |
| AKT1 | 16 |
| TP53 | 23 |

| **Supplementary Table S2. The 39 core nodes after the first screening and their topological parameters.** | |
| --- | --- |
| Node | Edge |
| AKT1 | 16 |
| BCL2 | 13 |
| BCL2L1 | 7 |
| BIRC5 | 4 |
| CASP3 | 14 |
| CASP8 | 7 |
| CAV1 | 6 |
| CCL2 | 9 |
| CCND1 | 12 |
| CDK1 | 7 |
| CDKN1A | 8 |
| CXCL10 | 8 |
| CXCL2 | 7 |
| CXCL8 | 10 |
| EGFR | 9 |
| ERBB2 | 5 |
| ESR1 | 15 |
| ESR2 | 5 |
| FOS | 11 |
| GSK3B | 5 |
| HSP90AA1 | 13 |
| ICAM1 | 7 |
| IFNG | 8 |
| IL1B | 12 |
| IL6 | 15 |
| MAPK1 | 13 |
| MAPK8 | 9 |
| MDM2 | 10 |
| MYC | 10 |
| NFKBIA | 6 |
| PRKCA | 5 |
| PRKCB | 4 |
| PTGS2 | 5 |
| RB1 | 4 |
| RELA | 11 |
| STAT1 | 8 |
| TNF | 15 |
| TP53 | 23 |
| XIAP | 8 |

| **Supplementary Table S3. The 10 hub nodes after the second screening and their topological parameters.** | |
| --- | --- |
| Node | Edge |
| TNF | 15 |
| CCND1 | 12 |
| RELA | 11 |
| CASP3 | 14 |
| IL6 | 15 |
| ESR1 | 15 |
| AKT1 | 16 |
| TP53 | 23 |
| BCL2 | 13 |
| HSP90AA1 | 13 |
